# Metabolomic–Metabolite Profiling: Progressive Insight and Biochemical Pathway in Crude Oil Waste Sludge Co-Composting Bioremediation

**DOI:** 10.3390/metabo16090605

**Published:** 2026-08-25

**Authors:** Onyedikachi Ubani, Veronica M. Ngole-Jeme

**Affiliations:** Department of Environmental Sciences, University of South Africa, Corner of Christiaan de Wet Road & Pioneer Avenue, Florida, Roodepoort 1709, Gauteng, South Africa; ngolevm@unisa.ac.za

**Keywords:** 16S rRNA, animal manure, biodegradation, co-composting, crude oil sludge-PAHs, GC-MS metabolomics-intermediates, hydrocarbon-degrading bacteria, KEGG pathways

## Abstract

**Background:** Crude oil refinery waste sludge (COWS) ranks among the most compositionally complex and ecotoxicologically hazardous industrial residues. Although bulk total petroleum hydrocarbon (TPH) and summed polycyclic aromatic hydrocarbon (PAH) removal are routinely reported, the metabolite-level biochemical fate of individual petrogenic compounds, spanning ring dihydroxylation, catechol cleavage, and entry into central carbon metabolism, remains largely unmapped under co-composting with diverse animal manures. **Objectives:** This study aimed to construct a metabolite-resolved, microbially anchored biochemical fate map of crude oil sludge during co-composting. Methods: Aerobic microcosms combining crude oil sludge, garden soil, and a wood-chip bulking agent were amended separately with poultry, horse, cow, or swine/pig manure alongside an unamended control and then incubated at 22 °C for 300 days. Analyses integrated untargeted gas chromatography-mass spectrometry (GC-MS) metabolomics, targeted PAH quantification (EPA Methods 3541/8270), 16S rRNA gene amplicon sequencing (Illumina MiSeq, V1–V3, paired-end 300 bp), physicochemical monitoring, and culture-dependent isolation, with National Institute of Standards and Technology (NIST) Mass Spectral library annotation. **Results:** GC-MS resolved 1169 metabolite features across 17 samples, comprising 538 annotated compounds within 11 chemical classes and 631 unknowns, of which 151 recurred in at least 10 samples. Petrogenic markers (n-alkanes C_14_–C_36_, hopanoids, steranes, and alkylated dibenzothiophenes) and ring-cleavage intermediates (2-hydroxyfluorene, 1,4-naphthoquinone, phenanthrene-methanol, benzenediols, butanedioic acid, fatty alcohols C_16_–C_20_) elucidated a four-stage degradation cascade consistent with Kyoto Encyclopedia of Genes and Genomes (KEGG) pathways map01220 and map00624. PAH mean-removal ranked swine/pig (88.0%) > horse (87.0%) > poultry (80.5%) > cow (79.1%) > control (68.2%). Sequencing recovered 2969 operational taxonomic units (OTUs) enriched in *Pseudomonas*, *Achromobacter*, *Stutzerimonas*, *Dietzia*, *Gordonia*, and *Mycobacterium*, with *Pseudomonas* dominating high-removal systems; respiration peaked at 18.7 mg CO_2_-C g^−1^ in poultry treatments. **Conclusions:** This work establishes a metabolite-resolved map linking hydrocarbonoclastic taxa to separate degradation steps. The co-occurrence of oxygenated PAH intermediates with decreasing parent PAH concentrations serves as an indicator of transformation processes and may assist in identifying potential residual-risk signals, thereby supporting remediation evaluation and process optimization.

## 1. Introduction

Accelerating industrialization has made crude oil refinery waste sludge (COWS) one of the most abundant, compositionally complex, and environmentally hazardous industrial wastes worldwide [1,2,3]. Generated throughout refinery operations, including desalting, atmospheric distillation, catalytic cracking, and wastewater treatment, COWS contains a heterogeneous mixture of aliphatic hydrocarbons, parent and alkylated polycyclic aromatic hydrocarbons (PAHs), resins, asphaltenes, heteroatom-containing compounds such as dibenzothiophenes, heavy metals, and inorganic salts [1,2,3]. Improper disposal of this waste facilitates the release of persistent contaminants into soil and groundwater, causing ecological disruption and posing substantial risks to human health through bioaccumulation and trophic transfer [4,5,6]. Among these contaminants, PAHs are of particular concern because their toxicity is primarily associated with metabolic activation to reactive epoxide intermediates that exhibit cytotoxic, mutagenic, and carcinogenic properties [7,8,9].

Composting, particularly co-composting with organic amendments, has emerged as an effective, scalable, and cost-efficient strategy for remediating petroleum-contaminated waste [10,11,12,13,14,15]. Organic amendments, such as animal manure create favourable conditions for hydrocarbon biodegradation by improving nutrient availability, carbon supply, and substrate aeration, thereby enhancing microbial activity, achieving 60–90% TPH removal, and enriching specialized microbial communities involved in petroleum degradation [15,16,17]. Despite these advances, remediation performance is commonly evaluated using bulk indicators, including total petroleum hydrocarbon (TPH) concentrations and the 16 US EPA priority PAHs, together with broad assessments of microbial community composition [18,19]. Although useful, these approaches provide limited insight into the biochemical pathways causing hydrocarbon transformation, the identity of intermediate metabolites, and their potential ecological risks.

Metabolomics offers a means of overcoming these limitations by providing a direct assessment of metabolic activity and biochemical transformation within complex environmental systems [20,21]. Unlike genomic or transcriptional approaches that infer functional potential, metabolomics captures the actual metabolic state of a system. In petroleum-contaminated matrices, gas chromatography-mass spectrometry (GC-MS) is particularly effective because of its compatibility with the volatility, structural diversity, and concentration range of hydrocarbons and their degradation products [22,23]. When integrated with KEGG pathway annotation, untargeted GC-MS metabolomics enables the reconstruction of hydrocarbon degradation pathways from parent PAHs and petrogenic compounds through ring-hydroxylated intermediates, catechol ortho- and meta-cleavage products, and tricarboxylic acid (TCA) cycle intermediates [20,21,22,23]. This pathway-level resolution provides a functional understanding of crude oil waste sludge bioremediation that cannot be achieved through conventional bulk measurements alone.

Nevertheless, important knowledge gaps remain. Oxygenated PAHs (OPAHs), which can form during incomplete aerobic degradation, are rarely monitored despite evidence that they may equal or exceed their parent compounds in toxicity and environmental persistence [24,25,26]. Similarly, conservative petrogenic biomarkers such as hopanes, steranes, and 25-norhopanes have rarely been incorporated into untargeted metabolomic investigations of composting systems, despite their value as indicators of petroleum origin and biodegradation intensity [27,28]. Furthermore, while hydrocarbon-degrading genera, including *Pseudomonas*, *Mycobacterium*, *Gordonia*, *Dietzia*, *Achromobacter*, and *Stutzerimonas*, are known to participate in PAH degradation, their links to specific metabolite signatures under co-composting conditions remain poorly understood. Comparative assessments of metabolomic responses among different manure amendments, including poultry, horse, cow, and swine manure, are also lacking, limiting evidence-based optimization of co-composting strategies.

Previous studies have highlighted the effectiveness of composting for hydrocarbon degradation but have not resolved the driving functional processes responsible for this degradation. Semple et al. [29] and Wu et al. [30] reported substantial PAH degradation during sludge composting without identifying intermediate metabolites. Likewise, Sayara et al. [14] and Antizar-Ladislao et al. [31] focused on TPH reduction and microbial succession, whereas Cébron et al. [32] linked microbial guilds to hydrocarbon mineralization without characterizing specific catabolic intermediates. Similarly, Lors et al. [33] and Fernández-Luqueño et al. [34] quantified targeted PAHs but did not incorporate untargeted metabolomics or KEGG-based pathway reconstruction.

Thus, this study integrated untargeted GC-MS metabolomics, targeted PAH quantification (EPA Methods 3541/8270), 16S rRNA amplicon sequencing (Illumina MiSeq V1–V3), physicochemical analyses, and culture-dependent microbial isolation within a unified framework. It was hypothesized that co-composting COWS with different manure amendments follows a common but amendment-specific biodegradation pathway, whereby primary petrogenic compounds, including n-alkanes (C_14_–C_36_), PAHs, hopanes, and steranes, are progressively transformed through ring-hydroxylated intermediates and catechol-cleavage pathways before entering central carbon metabolism. Variations in microbial community composition, enzymatic activity, and biosurfactant production are expected to influence the accumulation and turnover of these metabolites. Accordingly, this study aimed to: (i) characterize the GC-MS metabolomic profiles of control, oil-only, and manure-amended COWS co-composts after 300 days of incubation at 22 °C; (ii) reconstruct biodegradation pathways using KEGG modules map01220 and map00624, with emphasis on catechol-cleavage and phthalate-degradation routes; (iii) identify dominant hydrocarbon-degrading bacterial taxa through 16S rRNA sequencing; (iv) establish metabolite–microbe interaction networks; and (v) evaluate amendment-specific metabolomic signatures for process optimization and ecological risk assessment, particularly the occurrence of oxygenated intermediates in hydrocarbon depletion. Overall, this integrated approach advances the understanding of the functional processes governing COWS co-composting by linking metabolite transformations with microbial activity, thereby informing bioreactor optimization, regulatory risk assessment, and evidence-based amendment selection for large-scale bioremediation applications.

## 2. Materials and Methods

### 2.1. Sample Collection and Characterization

Crude oil sludge was obtained from a petroleum refinery in Durban, South Africa, and was subjected to characterization using an automated Soxhlet extraction system with dichloromethane as solvent and subsequently quantified via gas chromatography–mass spectrometry (GC-MS). To provide nutrient amendments, animal manures, including from cow, swine (pig), horse, and poultry, were collected from the University of Pretoria Farm (South Africa) and analysed for total organic carbon (TOC), nitrogen (N), and phosphorus (P) following established standard procedures [35,36]. In parallel, garden soil samples were collected at a depth of 20 cm from a farm in Tembisa, north of Kempton Park, East Rand, Gauteng Province, South Africa (26°1′0″ S, 28°14′0″ E). The soil was air-dried, homogenized and systematically characterized for physicochemical properties, including texture, pH, water-holding capacity (WHC), and elemental composition. Elemental profiling was performed using inductively coupled plasma optical emission spectrometry (ICP-OES; PerkinElmer Inc., Optima 5300 DV, Waltham, MA, USA) after digestion with concentrated aqua regia (HNO_3_: HCl, 1:3, *v*/*v*). Quantification relied on a 23-element Merck standard stock solution (Cat. No. 43843; 100 mg L^−1^, Merck KGaA, Darmstadt, Germany), from which ten calibration standards (0.2–8 mg L^−1^) were prepared in nitric acid. Filtrates from the digested soil samples were analysed under identical instrumental conditions to ensure accuracy and comparability [36,37].

### 2.2. Co-Compost Microcosm Setup

To establish the co-composting microcosms, 300 g of crude oil waste sludge (COWS) was dispersed in 400 mL of tetrachloromethane (CCl_4_, 99.55%) and homogenized with 1.0 kg of air-dried garden soil. The mixture was air-dried at room temperature until the solvent had fully evaporated, yielding ≈1.30 kg of oil-amended, air-dried soil. Air-dried wood chips were then incorporated at a soil-to-wood-chip ratio of 1:2 (*w*/*v*; 1.30 kg of oil-amended soil per 2000 mL of wood chips), producing a structured, air-dried matrix of 2.34 kg per microcosm. Each matrix was amended with 1.17 kg of air-dried cow, horse, pig, or poultry manure at a matrix-to-manure ratio of 2:1 (*w*/*w*), yielding a total microcosm mass of 3.51 kg. An unamended matrix (2.34 kg, no manure) served as the control. All masses are reported on an air-dried-weight basis, while a control setup without manure amendment was maintained for comparison. All treatments were prepared in triplicate and incubated for 300 days in aerated polyvinyl chloride (PVC) troughs (30 × 18 × 20 cm) under ambient laboratory conditions (22 °C, 100 kPa). Throughout the incubation period, moisture content was monitored weekly and carefully regulated between 50% and 80%. Temperature was measured by inserting a laboratory thermometer into the centre of the compost. Measurements were recorded daily during the first 7 days and every 3 days over the next two weeks. Thereafter, temperature was monitored weekly throughout the incubation period. The compost was turned every three days to maintain aeration and oxygen flow. All measurements were taken at midday to minimize temperature fluctuations [38,39,40]. The pH of the compost was determined monthly by analysing the aqueous extract using a calibrated pH meter (Crison Micro pH 2000™, Crison Instruments S.A., Alella, Barcelona, Spain), ensuring accurate and consistent monitoring of changes throughout the composting process. Microbial activity and compost maturity were assessed using a modified closed-jar CO_2_ evolution method [41,42], adapted from Atagana [43]. Duplicate moist compost samples (50 g) were incubated in sealed glass jars at room temperature. Each jar contained a suspended vial with 40 mL of 0.1 M NaOH to capture evolved CO_2_. After 3 days, the absorbed CO_2_ was precipitated with 2 mL of BaCl_2_ and quantified by titration with 2 M of HCl. Identical blank jars without compost were included as controls. Ash content was determined at the beginning and end stages of the experiment by weighing 10 g of each compost sample in a pre-weighed crucible, reweighing before and after combustion at 400 °C for 6 h in a furnace, and cooling in a desiccator to a constant weight to accurately quantify compositional changes. All chemicals and reagents used were of analytical grade and applied without further purification. The experimental treatment setup and sample codes are summarized in Table 1.

### 2.3. Culture-Dependent Isolation of Hydrocarbon-Degrading Bacteria

Hydrocarbon-degrading bacteria were enriched by inoculating 15 g of compost into 100 mL of mineral salt medium (MSM) supplemented with 10 mL of crude oil sludge as the sole carbon source. Cultures were incubated at 28 °C for 21 days under continuous agitation and subsequently subcultured to enhance adaptation. Serial dilutions (10^−6^–10^−8^) were plated on mineral salt agar (MSA). Morphologically distinct colonies were selected and purified on nutrient agar. Pure isolates were then grown in nutrient broth before genomic DNA extraction using the Quick g-DNA™ Kit (Zymo Research, Irvine, CA, USA) according to the manufacturer’s instructions.

### 2.4. DNA Extraction and Quality Assessment

Community DNA was extracted from 15 g of each compost sample suspended in 50 mL of phosphate-buffered saline (PBS). The suspensions were incubated overnight at 4 °C, homogenized by vortexing, and centrifuged at 12,000 rpm for 5 min at 4 °C. Total DNA was then isolated from the supernatant using the Faecal/Soil Total DNA™ Kit (Zymo Research Corporation, Irvine, CA, USA) according to the manufacturer’s protocol. Genomic DNA from compost samples and pure bacterial isolates were pooled for downstream analyses. DNA integrity was verified, and concentration was measured using a NanoDrop 2000 spectrophotometer (Thermo Scientific, Waltham, MA, USA). Only samples meeting the quality thresholds of A260/A280 = 1.8–2.0 and DNA concentrations of 20–150 ng/µL were used for subsequent analyses.

### 2.5. PCR Amplification, Library Preparation, and Sequencing

The 16S rRNA gene was amplified using a two-step nested PCR approach. The first reaction used universal primers 27F (5′-AGAGTTTGATCCTGGCTCAG-3′) and 1492R (5′-ACGGCTACCTTGTTACGACTT-3′) to target the near-full-length gene. A second nested PCR was then performed using primers 27F and 518R (5′-GTATTACCGCGGCTGCTGG-3′) to amplify the V1–V3 hypervariable regions for improved taxonomic resolution. PCR consisted of an initial denaturation at 95 °C for 10 min, followed by 32 cycles of denaturation at 95 °C for 30 s, annealing at 55 °C for 30 s, and extension at 72 °C for 1 min. A final extension was performed at 72 °C for 10 min. The amplicons were purified using the Zymo DNA Clean and Concentrator™ Kit (Zymo Research Corporation, Irvine, CA, USA). Sequencing libraries were prepared with Illumina-compatible adapters [44]. The libraries were sequenced as paired-end 300 bp reads (v3 chemistry) on the Illumina MiSeq platform (Illumina Inc., San Diego, CA, USA) at the University of South Africa, Florida Campus.

### 2.6. PAH Quantification by GC-MS

Polycyclic aromatic hydrocarbons (PAHs) were extracted from compost samples using automated Soxhlet extraction with dichloromethane according to EPA Method 3541 [45,46]. PAH concentrations were subsequently determined by GC-MS (Agilent 7890 GC coupled to a 5975C MSD) following EPA Method 8270. Calibration curves (10–50 ppm) were prepared from a 1000 ppm stock standard (Restek Cat. No. 8270-1). Chromatographic separation was performed using helium as the carrier gas at 1 mL/min with a 1 μL splitless injection. The oven temperature was held at 40 °C for 1 min, increased at 10 °C/min to 310 °C, and held for 5 min. Method performance was assessed using certified reference standards [47,48].

Untargeted metabolite profiling was performed on the same GC-MS platform. Raw chromatographic data were processed through automated deconvolution and peak detection workflows. Metabolites were assigned by spectral matching against the NIST Mass Spectral Library (version 2017) and an in-house petroleum-specific database. Metabolite annotations were reported according to Metabolomics Standards Initiative (MSI) guidelines as Level 1 (reference standard matched), Level 2 (putatively identified; similarity index ≥800), or Level 3 (putatively characterized compound class) [49].

### 2.7. Integrated Degradation Pathway, Biosurfactant Production, and Whole-Sludge Metabolic Mapping of Crude Oil Refinery Waste

A time-course experiment was conducted to assess the potential contribution of endogenously produced biosurfactants to COWS degradation. The study examined whether biosurfactant production could enhance hydrocarbon bioavailability, support metabolite formation, and potentially serve as an alternative to exogenous Tween 80. Cell-free supernatants were collected every 24 h over a 7-day incubation period. Surface tension, emulsification index (E_24_), biosurfactant yield, and biomass accumulation were measured at each sampling point. Biosurfactant activity was further evaluated using oil-displacement, drop-collapse, and emulsification assays. These complementary assays were used to assess the capacity of the microbial consortium to improve hydrocarbon bioavailability through the production of surface-active compounds.

### 2.8. Bioinformatic Analysis

Raw sequencing reads were subjected to rigorous quality control using ngsShoRT v2.1. [50]. This systematically trimmed low-quality bases, removed ambiguous nucleotides, and filtered out PCR artefacts to ensure data integrity. Chimeric sequences were subsequently detected and eliminated using the UCHIME algorithm v4.2.40 [50,51], thereby refining dataset accuracy. The resulting high-quality reads were processed within the Mothur pipeline (v1.40.0) [52], where they were aligned against the SILVA 16S rRNA reference database (releases 128 and 132) [53] to facilitate precise phylogenetic placement. Sequences were then clustered into operational taxonomic units (OTUs) at a 97% similarity threshold, reflecting species-level resolution. Taxonomic assignments were performed using a Naïve Bayesian classifier with an 80% confidence cut-off [54], and representative OTUs were further compared against the NCBI nucleotide database through BLASTn searches (BLAST+ version 2.14.0) [55] to ensure annotation reliability. To characterize community diversity, alpha diversity indices, including Shannon–Weaver and Chao1, were calculated at a genetic distance of 0.03, while Good’s coverage estimator was applied to evaluate sequencing depth and ensure adequate sampling of microbial diversity.

### 2.9. Predicted Functional Profiling

To elucidate the catabolic functional potential of the co-compost microbial communities, the 16S rRNA amplicon sequence variant (ASV) features were subjected to metagenome content prediction using PICRUSt2 (version 2.5.2), whereby ASVs were phylogenetically placed into a reference tree, and KEGG Orthology (KO) gene-family abundances were predicted and normalized by predicted 16S rRNA copy number to correct for gene copy number bias. Prediction reliability was rigorously assessed using the weighted Nearest Sequenced Taxon Index (NSTI), with mean values ranging from 0.05 to 0.16 across all samples, well within the conventionally accepted confidence interval, and features exceeding an NSTI threshold of 2.0 were excluded from downstream analysis to maintain high-confidence estimates. These low NSTI values are consistent with communities dominated by well-characterized, genomically represented genera such as *Pseudomonas*, *Achromobacter*, and allied taxa, collectively affirming the reliability and validity of the inferred functional profiles [56,57].

Predicted KO abundances were mapped to two curated KEGG pathways: degradation of aromatic compounds (map01220) and polycyclic aromatic hydrocarbon degradation (map00624) [58,59]. The mapped functions were then grouped into four stages of the biodegradation process. These stages included ring activation, ring cleavage, lower-pathway processing, and aliphatic oxidation. The classification was designed to align with the degradation patterns inferred from metabolomic profiling. Per-stage KO abundances were standardized as counts per million to enable robust, internally consistent cross-treatment comparisons, thereby bridging community composition data with experimentally validated metabolite evidence and reinforcing the genotype-to-phenotype framework established across the study.

### 2.10. Data Processing and Statistical Analysis

Metabolite data were compiled into a feature table containing peak identities, retention times, characteristic mass-to-charge (*m*/*z*) ratios, peak areas, and NIST spectral similarity indices for all 17 samples. Features with similarity indices ≥600 were classified as identified metabolites, whereas those below this threshold were designated as unknown. Two datasets were generated for subsequent analyses: a binary presence–absence matrix and a quantitative peak-area matrix. All statistical analyses were performed in R (v4.x) using the vegan and stats packages.

Descriptive statistics were used to evaluate metabolite richness, identification success, and chemical-class distribution. Metabolite occurrence frequencies were assessed to identify ubiquitous features (in ≥10 of 17 samples), treatment-specific signatures, and unique compounds. Based on NIST annotations, metabolites were categorized into n-alkanes, parent PAHs, alkylated PAHs, sulfur-containing heterocyclic PAHs, oxygenated PAH intermediates, phthalate esters, hopanoids/steranes, fatty acids and alcohols, organic acids, phenolics, and nitrogen-containing compounds.

Treatment effects on overall metabolite composition were evaluated using permutational multivariate analysis of variance, applied to Bray–Curtis dissimilarities derived from the peak-area matrix and Jaccard dissimilarities derived from the presence–absence matrix. Homogeneity of multivariate dispersion, a key assumption, was assessed before interpretation. Patterns in metabolite composition were visualized through unsupervised principal component analysis (PCA), with treatment groups and metabolite classes included as categorical descriptors.

For individual metabolites, data were first tested for normality using the Shapiro–Wilk test and for homogeneity of variance using Levene’s test. Because peak-area data did not satisfy parametric assumptions, differences among treatments were assessed using the Kruskal–Wallis test followed by Dunn’s post hoc test for pairwise comparisons. Associations between metabolite occurrence and treatment were examined using Fisher’s exact test. To control multiple comparisons, *p*-values were adjusted using the Benjamini–Hochberg false-discovery-rate (FDR) procedure, and adjusted q-values are reported. Statistical significance was defined at a two-tailed α level of 0.05, with metabolites considered significantly differentially represented when q < 0.05.

Functional predictions were conducted through pathway-based analysis by mapping annotated metabolites to the Kyoto Encyclopedia of Genes and Genomes (KEGG) degradation of aromatic compounds superpathway (map01220) and PAH degradation pathway (map00624), complemented by MetaCyc pathways associated with catechol meta-cleavage and phthalate degradation [60,61]. This integrative approach enabled the linkage of predicted metabolites to putative hydrocarbon degradation processes and broader carbon metabolic pathways.

## 3. Results

### 3.1. Sample Characterization and Physicochemical Evolution During Co-Composting

In the 300-day co-composting experiment, integrated physicochemical, nutritional, and biological signals converged to describe a coherent progression of crude oil sludge transformation. The sandy loam soil matrix (pH 5.56; 13.01 mg kg^−1^ organic carbon; 32.62% WHC) offered limited intrinsic biodegradation capacity (Table 2), but amendment with pig, poultry, cow, and horse manure introduced distinct nutrient regimes that governed microbial activity and hydrocarbon metabolism.

Pig manure exhibited the highest water-extractable organic carbon concentration (904 ± 84 mg kg^−1^), followed by poultry manure (277 ± 63 mg kg^−1^), cow manure (109 ± 8 mg kg^−1^), and horse manure (81 ± 3 mg kg^−1^), while extractable organic nitrogen remained comparatively uniform (49.2–54.9 mg kg^−1^; <12% variation), establishing a stable nitrogen background. In contrast, extractable phosphorus stratified sharply into a high-P tier (poultry: 254 ± 14 mg kg^−1^; pig: 252 ± 29 mg kg^−1^) and a low-P tier (horse: 50 ± 2 mg kg^−1^; cow: 46 ± 8 mg kg^−1^)—a five-fold separation with no overlap. Carbon availability followed the same broad grouping (pig, poultry > cow, horse), although pig’s carbon pool was itself markedly elevated relative to poultry, indicating that the high-C–high-P and low-C–low-P designations reflect co-elevated, independently governed nutrient pools rather than a matched C:P stoichiometric ratio across the two manure pairs (Table 3). These gradients critically shaped compost transformations and biodegradation potential.

**Figure 1:**
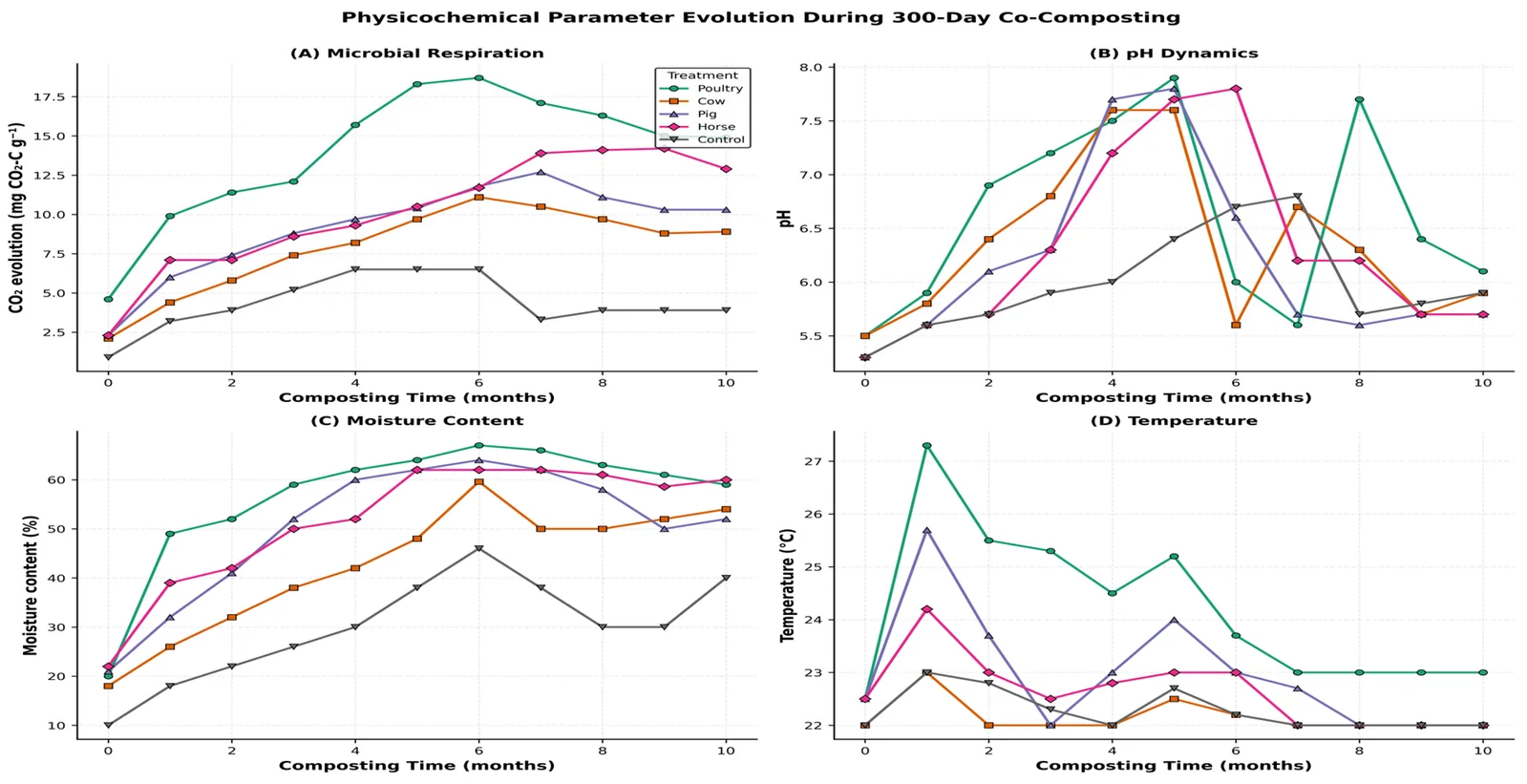
Physicochemical parameter evolution. Time course of (**A**) CO_2_ respiration, (**B**) pH, (**C**) moisture, and (**D**) temperature over ten months: Poultry and horse amendments sustained the highest respiration (peaking at 18.7 mg CO_2_-C g^−1^), the most robust signal of active microbial mineralization. The pH trajectory towards neutrality and stable moisture shows that maturation proceeded under conditions favourable for hydrocarbon-degrading consortia.

Figure 1 documents the ten-month physicochemical evolution across all manure-amended treatments and the unamended control, collectively revealing the biological and chemical environment that drove PAH mineralization. Microbial respiration (Figure 1A), the most direct indicator of active organic matter transformation, was highest in poultry-amended treatment (4.6 ± 0.01 to 18.7 ± 0.12 mg CO_2_–C g^−1^), followed by horse (2.3 ± 0.1 to 12.9–14.2 ± 0.1), pig (2.3 ± 1.0 to 12.7 ± 0.1), and cow (2.1 ± 0.1 to 11.1 ± 0.1) amendments, all showing vigorous decomposer activity and progressive hydrocarbon degradation. Meanwhile, the unamended control peaked modestly at 6.5 ± 0.1 before declining, highlighting the indispensability of organic nutrient supplementation for activating indigenous microbial consortia. pH trajectories (Figure 1B) consistently shifted from acidic baselines (5.3 ± 0.1) towards neutral-to-alkaline values at maturation (7.8 ± 0.1), with the poultry system peaking at 7.9, values corresponding precisely to the reported pH optima of hydrocarbon-degrading consortia. This amendment-driven alkalization, mediated by organic buffering capacity and accumulation of alkaline aerobic metabolites, enhanced enzymatic efficiency, broadened degrader ecological niches, and facilitated organically bound phosphorus solubilization, while the unamended control exhibited only minimal pH variation. Moisture content (Figure 1C) reached the aerobically optimal 50–80% range across all amended treatments, being most pronounced in the poultry, pig, and horse systems and sustaining the oxygen diffusion, aqueous film continuity, and substrate–microorganism contact essential for enzymatic catalysis, whereas the unamended control remained below 46%, a threshold consistently associated with suppressed microbial metabolic rates. Temperature profiles (Figure 1D) further supported these stimulatory trends: poultry amendment induced the strongest thermogenic response (22.5–27.3 °C), pig and cow treatments reached up to 25.7 °C, and horse amendment produced a sustained elevation to 24.2 °C, all remaining within the mesophilic range, which is optimal for hydrocarbon-degrading bacteria that dominate organic-rich amended soils because of the composting size. Meanwhile, the unamended control fluctuated passively near ambient conditions (~22–23 °C), indicating the absence of endogenous thermal generation. Notably, the ash content remained constant throughout the experimental period (Table 4). This finding indicates that the observed mass reductions resulted from genuine organic matter mineralization rather than physical redistribution or inorganic dissolution. It also highlights the synergistic interaction of respiration, pH, moisture, and temperature as key drivers of sustained bioremediation efficacy. Biological activity, indexed by CO_2_ evolution, provided the clearest evidence of microbial stimulation and progressive degradation.

This metabolic intensity aligned with the nutrient gradients and coincided with measurable reductions in structurally diverse PAHs, including naphthalene, anthracene, pyrene, chrysene, benzo[a]pyrene, and indeno(1,2,3-cd) pyrene, demonstrating that each amendment created conditions conducive to hydrocarbon biodegradation, albeit with treatment-specific efficiencies. Overall, the system revealed a tightly coupled physicochemical–biological nexus in which carbon and phosphorus availability, under stable nitrogen conditions, orchestrate microbial respiration, enzyme activation, and progressive pollutant transformation.

### 3.2. Microbial Community Structure Mediated Manure-Driven PAH Removal

**Figure 2:**
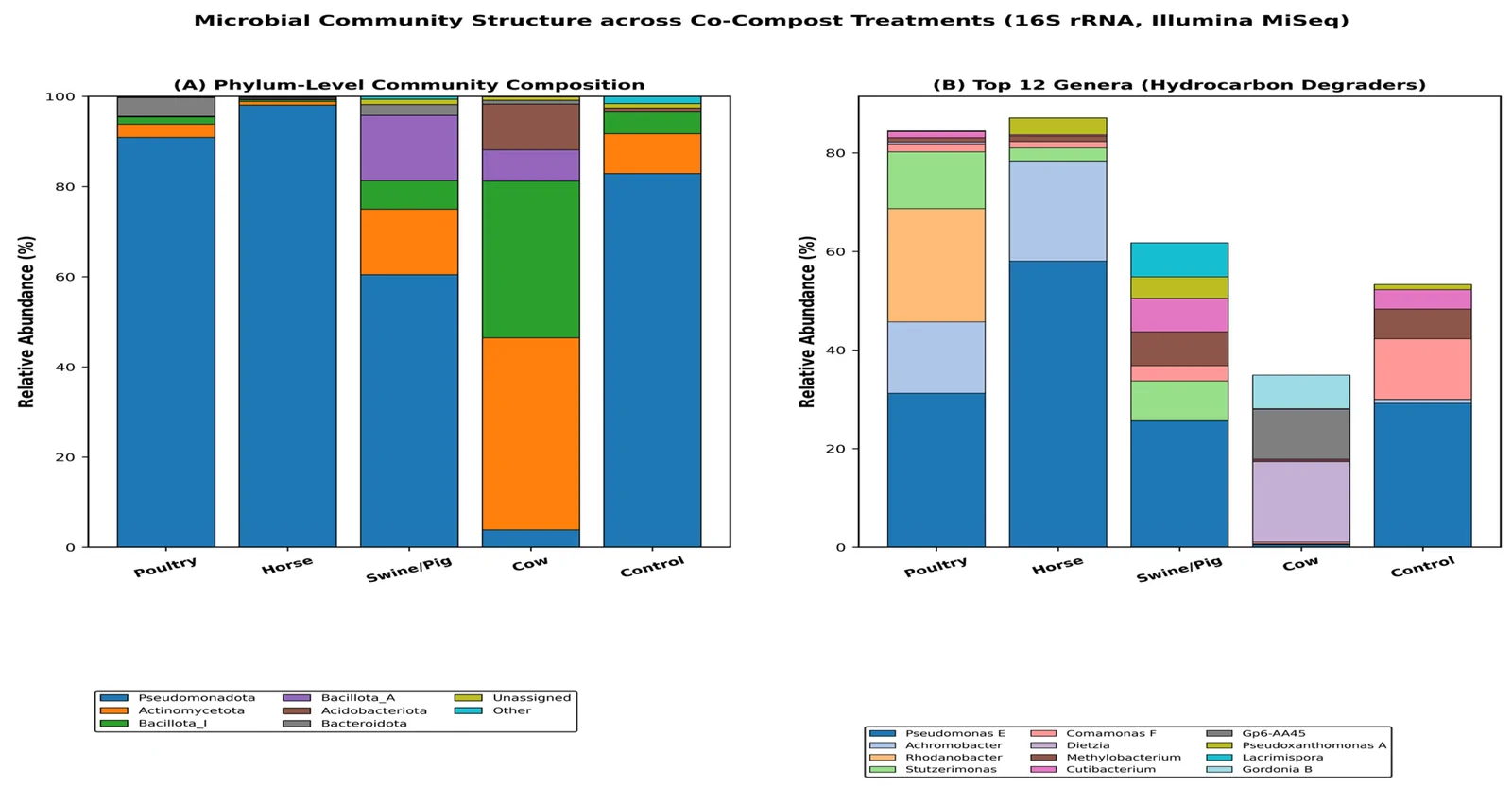
Microbial community composition: Relative abundance at (**A**) phylum and (**B**) genus levels from 16S rRNA sequencing: *Pseudomonadota* dominated manured treatments (up to 98% in horses), the phylum housing the most known hydrocarbonoclastic taxa, whereas cow manure uniquely enriched *Actinomycetota* (*Dietzia*, *Gordonia*, *Mycobacterium*). This taxonomic divergence provides the biological basis for amendment-specific degradation signatures.

**Figure 3:**
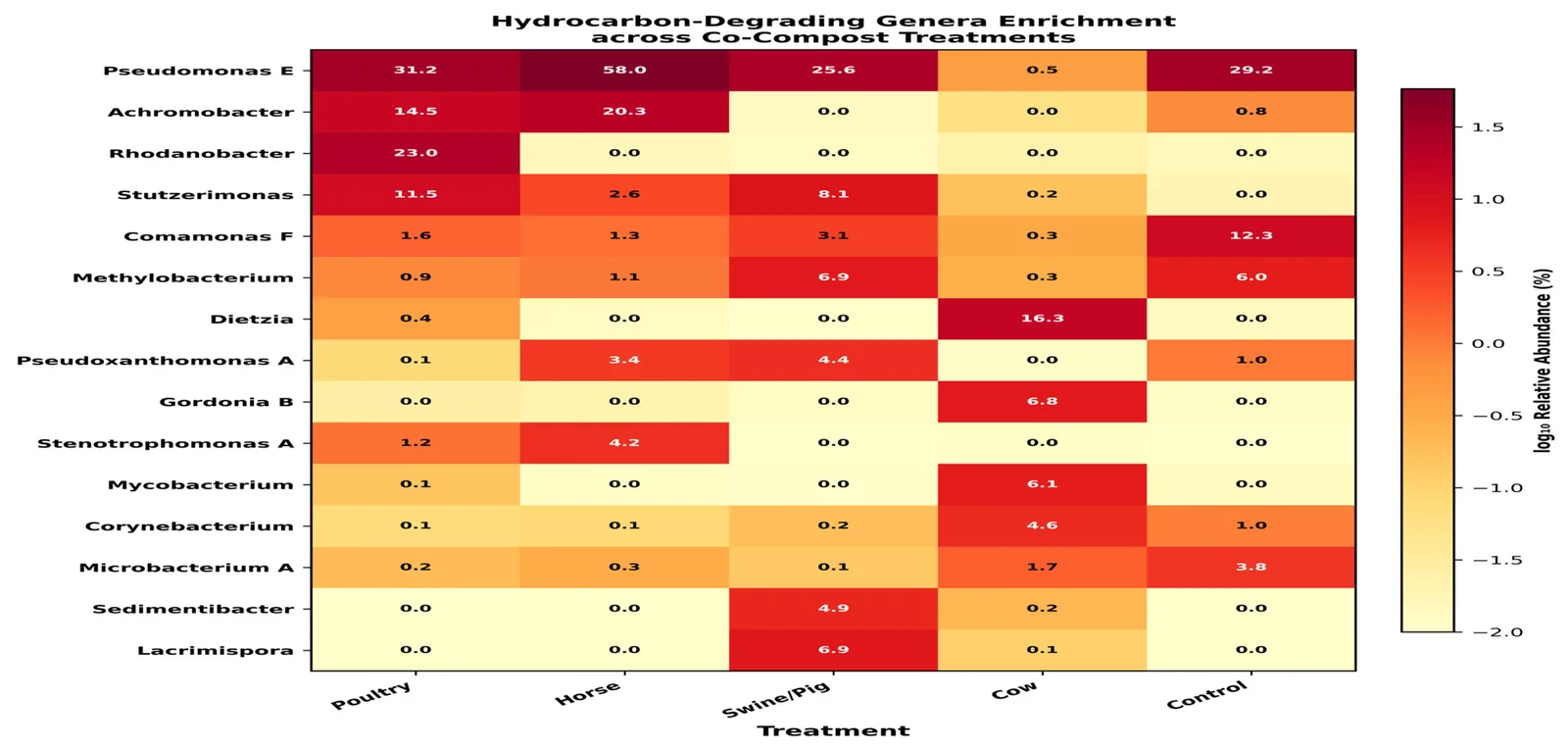
Hydrocarbon-degrader enrichment heatmap. Log-scaled relative abundance of 15 hydrocarbon-degrading genera across treatments: The heatmap exposes a clear division of labour: *Pseudomonas* and *Achromobacter* drive the horse and poultry systems, while an actinobacterial guild (*Dietzia*, *Gordonia*, *Mycobacterium*, *Corynebacterium*) is concentrated in cow manure. The presence of complementary degraders explains why multiple amendments achieve high removal through distinct microbial routes.

Sequencing of the 16S rRNA gene resolved 2969 OTUs spanning 15 phyla and 288 genera, identifying the microbial agents that drove the observed chemistry. *Pseudomonadota* dominated every manured community, climbing to 98% relative abundance under horse amendment, whereas cow manure established a distinct actinobacterial niche enriched in *Dietzia*, *Gordonia*, and *Mycobacterium* (Figure 2 and Figure 3).

**Figure 4:**
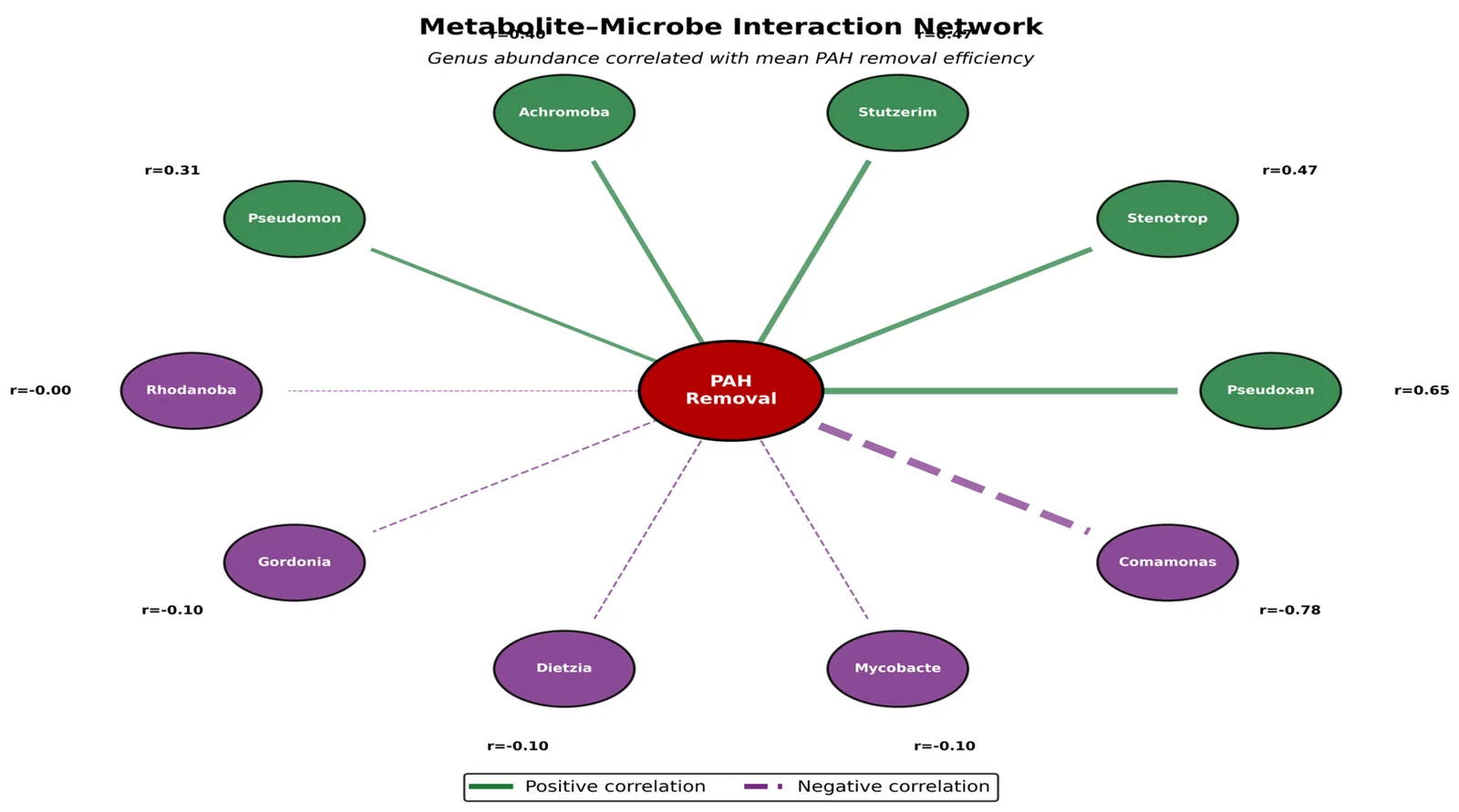
Metabolite–microbe interaction network: Correlation network linking degrader abundance to mean PAH removal. Green solid edges are positive, while purple dashes are negative; edge width is scaled to correlation magnitude. *Pseudoxanthomonas*, *Stutzerimonas*, and *Achromobacter* correlated positively with removal, implicating them as primary functional drivers, whereas the negative association of *Comamonas* suggests a successional or secondary-consumer role. The network translates co-occurrence into testable functional hypotheses.

The degrader heatmap revealed a clear division of metabolic roles: *Pseudomonas*, *Achromobacter*, *Stutzerimonas*, and *Rhodanobacter* were associated with the high-removal poultry and horse systems, whereas the actinobacterial guild was associated with cow treatment through a potentially distinct catabolic route. The interaction network indicated positive associations between *Pseudoxanthomonas*, *Stutzerimonas*, *Achromobacter*, and PAH-removal efficiency, suggesting that these genera contributed to hydrocarbon transformation processes within the composting systems (Figure 4).

In the construction of the network in Figure 4, treatment-level relative abundances of ten candidate hydrocarbon-associated genera (*Pseudomonas*, *Achromobacter*, *Stutzerimonas*, *Dietzia*, *Gordonia*, *Mycobacterium*, *Rhodanobacter*, *Comamonas*, *Pseudoxanthomonas*, and *Stenotrophomonas*) were compared with the mean PAH removal efficiency of each treatment. Relative abundance is expressed as the percentage of total assigned reads per sample, and abundance patterns across the five treatments (poultry, horse, swine, cow, and control; *n* = 5) were related to treatment-level PAH-removal efficiencies using Pearson correlation coefficients (r). Genera exhibiting no variation across treatments were excluded.

**Figure 5:**
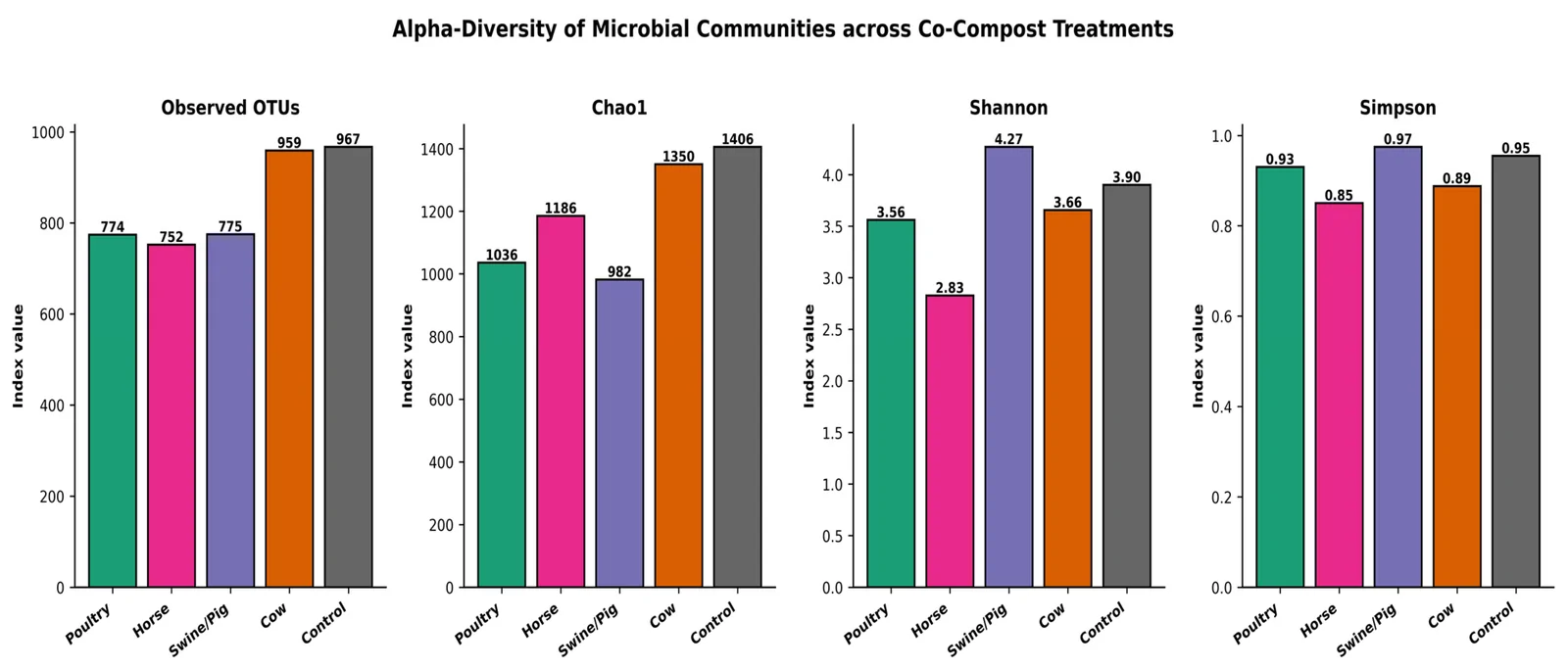
Alpha-diversity indices. Observed OTUs, Chao1 richness, and Shannon and Simpson diversity per treatment. Pig manure supported the most even, diverse community (Shannon 4.27; Simpson 0.975), consistent with its top-ranked PAH removal and supporting the principle that functionally redundant, diverse consortia confer robust degradation. Horse manure, although lower in evenness, achieved high removal through the dominance of a few highly active degraders, an alternative ecological strategy.

Each genus-removal relationship was represented as a network edge, with colour and line style indicating the direction of the association (green solid, positive; purple dashed, negative) and edge width being proportional to the absolute correlation coefficient (|r|). Given the limited number of treatment-level observations and the exploratory nature of the analysis, no correlation threshold or statistical significance filter was applied. Accordingly, the network was interpreted as a descriptive, hypothesis-generating visualization of potential associations between microbial taxa and remediation performance rather than evidence of direct functional contributions or statistically supported interactions. Finally, the alpha-diversity profile reconciled two equally successful ecological strategies (Figure 5 and Table 5): pig manure secured top-ranked removal through a highly even, diverse consortium (Shannon 4.27), whereas horse manure matched that performance by concentrating activity in a few dominant specialists.

### 3.3. PAH Quantification by GC-MS

**Figure 6:**
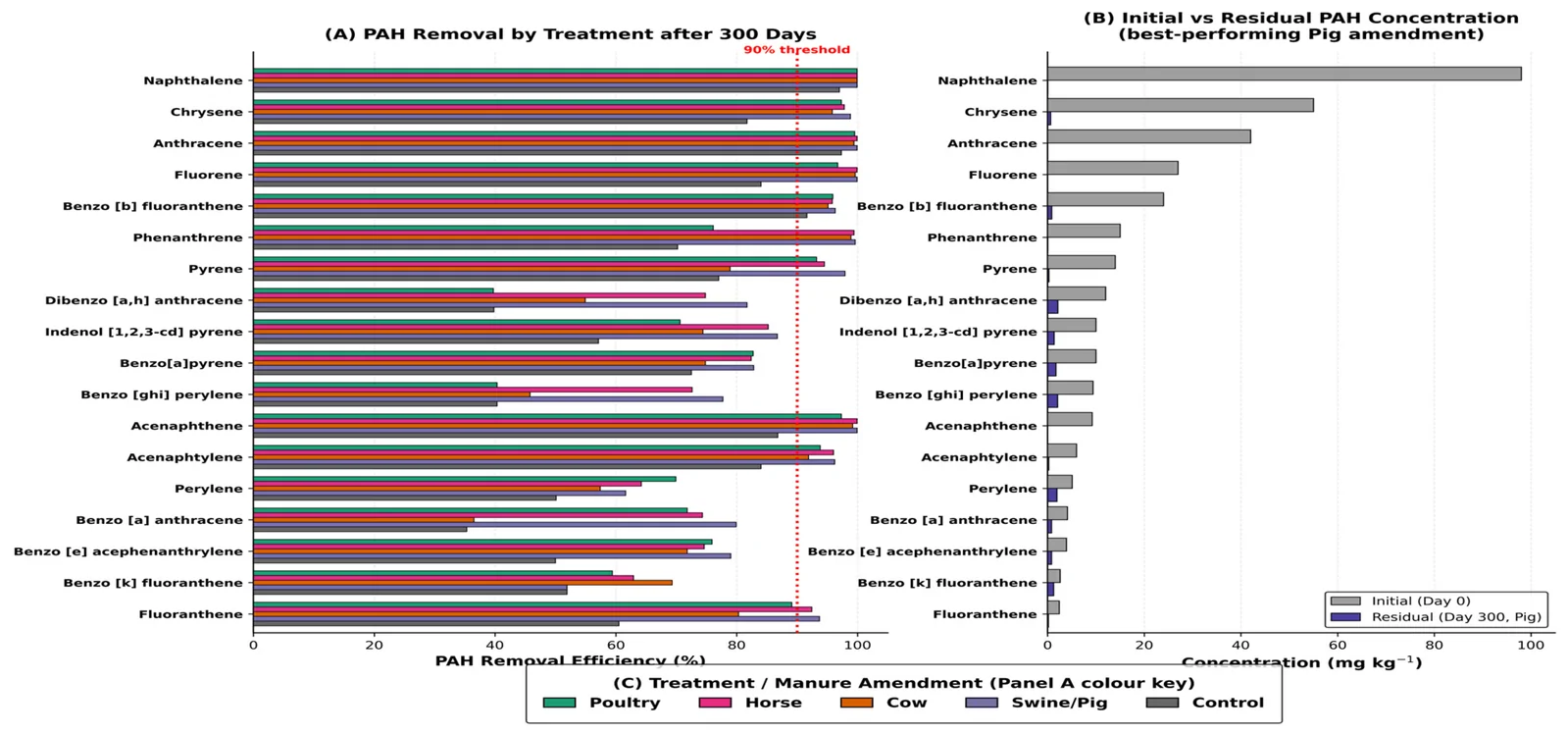
Polycyclic aromatic hydrocarbons (PAH) degradation kinetics in co-composting. (**A**) Removal efficiency of 18 PAHs by treatment; the red dashed line marks the 90% threshold. (**B**) Initial versus residual concentrations under the best-performing pig amendment: Low-molecular-weight PAHs (naphthalene, anthracene, fluorene) were almost completely removed (>99%) across manured treatments, whereas high-molecular-weight congeners (dibenzo[a,h]anthracene, benzo[ghi]perylene) remained comparatively recalcitrant, most so in the control (35–40%). The consistent superiority of pig and horse amendments over the control attest quantifiably to the biostimulatory value of manure.

The chemical signature of microbial activity was clearly reflected in the targeted PAH analysis (Figure 6 and Table 6), where all manure-amended treatments outperformed the control according to their mean removal ($\bar{x}$) ($\bar{x}$ = 68.2%). Pig ($\bar{x}$ = 88.0%) and horse ($\bar{x}$ = 87.0%) amendments achieved the highest overall reductions, followed by those of poultry ($\bar{x}$ = 80.5%) and cow ($\bar{x}$ = 79.1%), highlighting the decisive influence of amendment type on degradation efficiency. Low-molecular-weight PAHs (2–3 rings), including naphthalene, acenaphthene, fluorene, and anthracene, were almost completely removed, frequently exceeding 99%, whereas high-molecular-weight congeners exhibited more complex, treatment-dependent responses. Notably, recalcitrant compounds such as dibenzo[a,h]anthracene and benzo[ghi]perylene persisted in the control and poultry systems but were substantially reduced under pig and horse amendments, highlighting enhanced biodegradation precisely where resistance is greatest. Integrated performance assessment further confirmed effective PAH mineralization under co-composting conditions that simulate diffusion-limited, aged sludge environments characterized by sorption, sequestration, hydrophobic partitioning, and adsorption to soil organic matter. After 300 days, ring-class removal patterns demonstrated sustained degradation across increasing aromatic complexity, extending beyond readily degradable fractions into the persistent 4–6 ring PAHs that largely dictate toxicity and environmental persistence. Compound-specific reductions ranged from modest removal—36.5% for benzo[a]anthracene (4-ring) in cow, 39.7% for dibenzo[a,h]anthracene (5-ring) in poultry, and 40.3% for benzo[ghi]perylene (6-ring) in poultry—to near-complete transformation, reaching 98.8% for chrysene (4-ring), 96.3% for benzo[b]fluoranthene (5-ring), and 86.7% for indeno [1,2,3-cd]pyrene (6-ring) for pig-amended systems, according to GC-MS detection limits. Within the high-molecular-weight fraction, four-ring PAHs were removed most efficiently overall, although benzo[a]anthracene consistently showed lower degradation across treatments. Importantly, five- and six-ring PAHs, classified as priority Group 1 carcinogens and typically resistant due to extreme hydrophobicity and low aqueous solubility, also exhibited substantial reductions, with pig manure amendment revealing the highest individual removal performance across this most recalcitrant class.

### 3.4. Predicted Functional Metagenome Potential of the Multi-Stage Hydrocarbon Degradation Pathway Across Co-Compost Treatments

**Figure 7:**
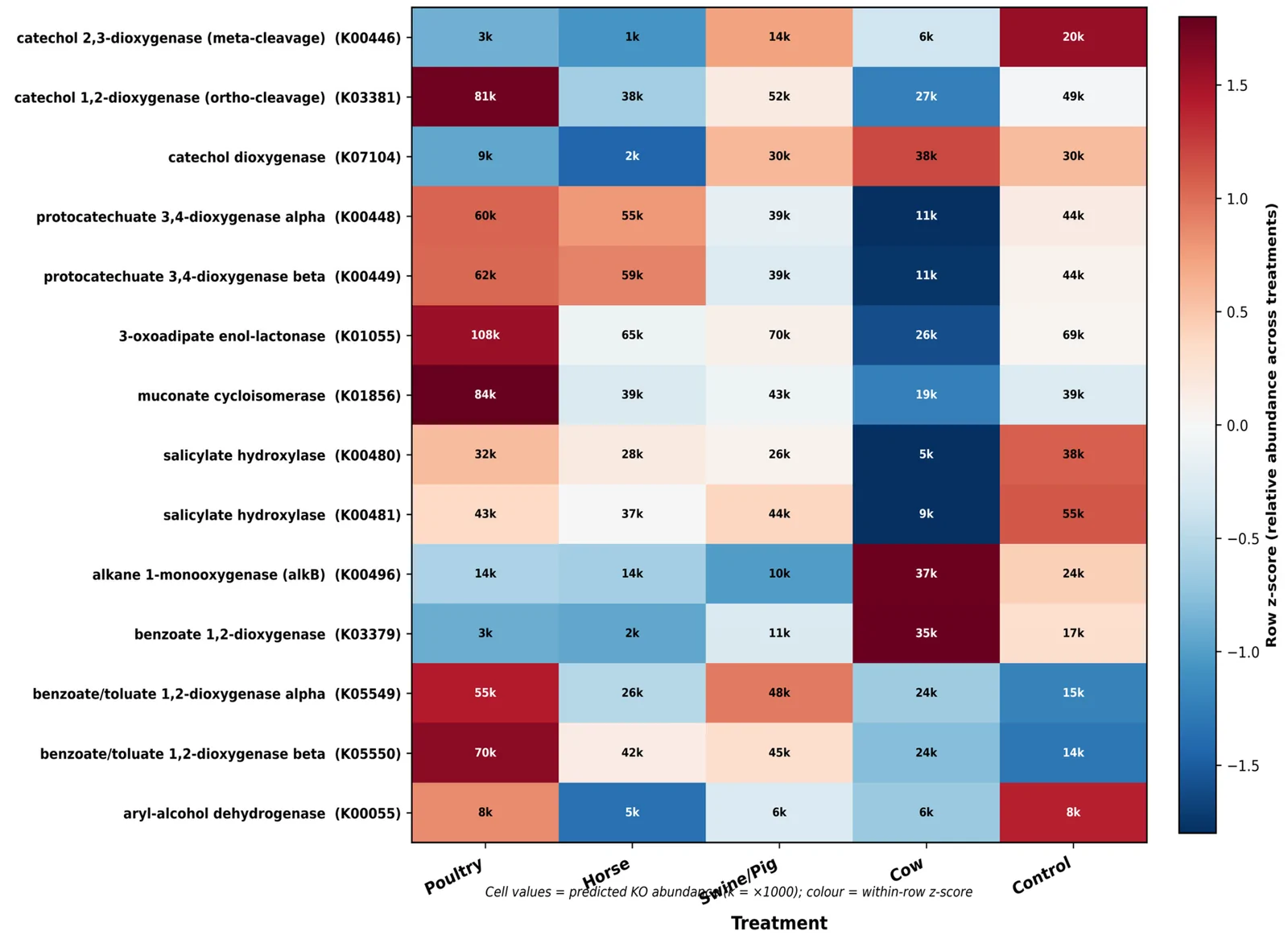
Predicted catabolic gene abundance heatmap. Heatmap showing the abundance of 14 PICRUSt2-predicted KEGG Orthologs (KOs) associated with hydrocarbon and aromatic compound degradation across treatments. Cell values indicate predicted KO abundance (×10^3^). Colours represent row-wise z-scores and highlight relative enrichment patterns among treatments.

To assess the functional potential associated with the observed metabolite patterns, PICRUSt2 was used to infer metagenomic profiles from 16S rRNA amplicon data. A total of 7160 KEGG Orthologs (KOs) were predicted across all treatments. These KOs spanned multiple stages of aerobic aromatic hydrocarbon degradation (Table 7 and Figure 7).

Predicted functional profiles varied among amendment types. Poultry-amended treatments showed higher predicted abundances of catechol 1,2-dioxygenase (K03381), muconate cycloisomerase (K01856), and 3-oxoadipate enol-lactonase (K01055). These enzymes are associated with ortho-cleavage and downstream aromatic degradation pathways. In contrast, cow-manure treatments showed higher predicted abundances of alkane 1-monooxygenase (alkB; K00496) and benzoate 1,2-dioxygenase (K03379). These functions are commonly linked to alkane and benzoate transformation pathways.

Ring-cleavage functions were predominantly represented by the ortho-cleavage pathway. Predicted abundance of catechol 1,2-dioxygenase (K03381) was highest in poultry-amended treatments (81,220) and lowest in cow-manure treatments (27,394). In contrast, catechol 2,3-dioxygenase (K00446), which is associated with meta-cleavage, showed higher predicted abundances in unamended control and swine-manure treatments. This pattern suggests treatment-specific differences in the distribution of predicted ring-cleavage functions.

Predicted abundances of protocatechuate 3,4-dioxygenase (K00448/K00449), muconate cycloisomerase (K01856), and 3-oxoadipate enol-lactonase (K01055) were consistently higher in poultry- and horse-manure treatments. These enzymes are associated with the β-ketoadipate pathway and may contribute to the processing of aromatic degradation intermediates into central metabolic pathways.

Notably, alkane 1-monooxygenase (alkB; K00496) and benzoate 1,2-dioxygenase (K03379) reached their highest predicted abundances in the cow-amended treatments, with 37,264 and 34,540 counts, respectively. These patterns aligned with the distinctive actinobacterial community structure identified by 16S rRNA sequencing, suggesting an enrichment of predicted hydrocarbon-degradation functions in these treatments.

**Figure 8:**
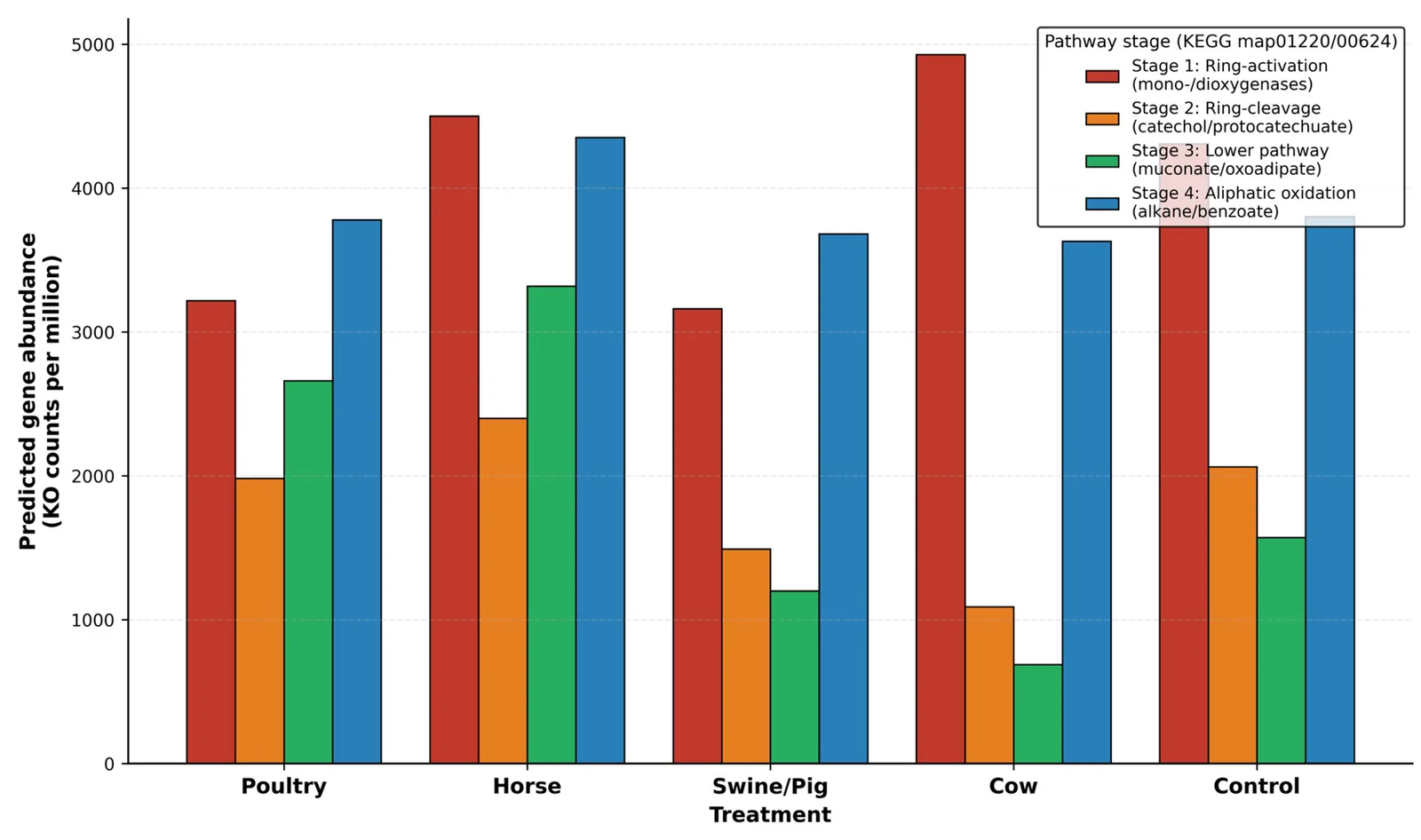
Predicted functional capacity across biodegradation pathway stages. Grouped bar chart showing PICRUSt2-predicted gene abundances (KO counts per million) mapped to four biodegradation stages: ring activation, ring cleavage, lower-pathway processing, and aliphatic oxidation. Values are presented for each treatment and represent pathway-stage-level functional predictions.

When grouped by degradation stage (Figure 8), all treatments exhibited predicted functional potential across four key processes: ring activation, ring cleavage, lower-pathway processing, and aliphatic oxidation. However, horse- and poultry-amended systems displayed the most balanced distribution of predicted functions across these stages, indicating that amendment type may influence both microbial community composition and the relative representation of hydrocarbon-degradation pathways.

Collectively, the predicted functional profiles were broadly consistent with the metabolomics-derived degradation patterns. These findings suggest that differences in manure amendment may influence the distribution of microbial metabolic capabilities associated with hydrocarbon transformation, although functional activity cannot be inferred directly from taxonomic composition or predicted gene abundance alone.

### 3.5. Overview of the Metabolomic Landscape

Untargeted GC-MS profiling of 17 co-compost samples detected 1169 unique metabolite features. Of these, 538 (46.0%) were annotated using the NIST spectral library, while 631 (54.0%) remained unassigned. Feature counts ranged from 145 in the soil-only control (SO) to the analytical detection limit of 300 features. Of the 17 samples, 14 reached this limit, indicating greater molecular complexity in petroleum-amended treatments.

The non-contaminated controls, acetone (ACE; 148 features) and soil-only (SO; 145 features), exhibited substantially lower feature richness. However, both showed annotation rates above 91%. In SO, the detected metabolites were largely common soil-derived compounds, including amino acids, organic acids, sugars, fatty acids, and sterols, which are well represented in spectral libraries [62,63,64]. In ACE, most signals originated from procedural and instrument-related sources, such as polysiloxane fragments, plasticizers, antioxidant stabilizers, and derivatization reagent carry-over [65,66].

The similar features observed in ACE and SO defined the lower range of molecular complexity in this study. This baseline provided a useful reference for interpreting the substantially higher diversity detected in petroleum-amended treatments. In contrast, all crude-oil-amended samples reached the 300-feature detection threshold. Annotation rates ranged from 38.0% in H3 (horse manure replicate 3) to 74.3% in PO1 (poultry manure replicate 1). Poultry manure treatments consistently produced the highest annotation rates (64.7–74.3%). This pattern suggests that these treatments generated transformation products with greater similarity to compounds represented in available reference libraries.

**Figure 9:**
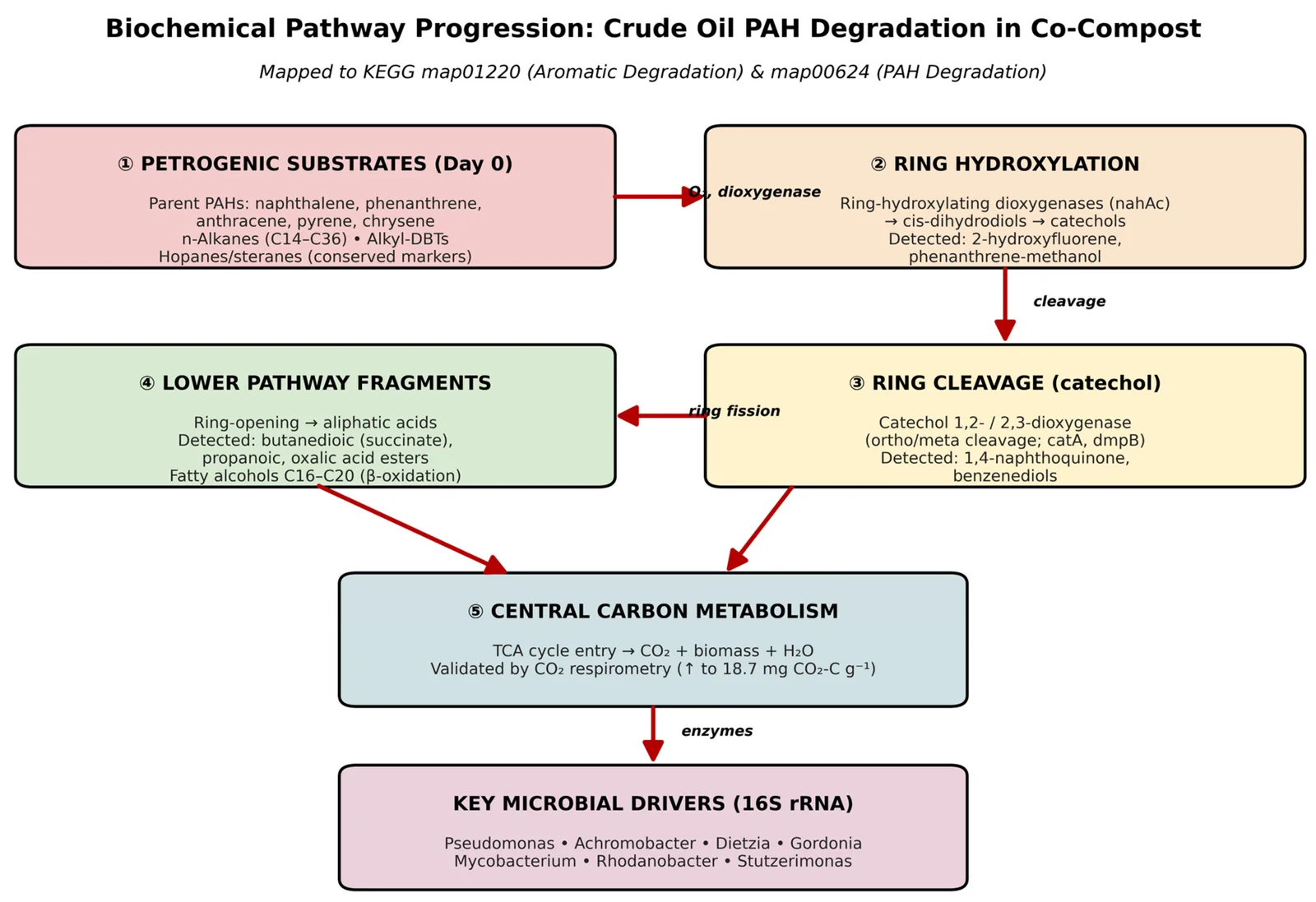
KEGG biochemical pathway progression. Five-stage degradation cascade mapped to KEGG map01220 and map00624, with detected intermediates and microbial drivers annotated. Metabolites detected in the GC-MS atlas populate every stage of the canonical aerobic aromatic degradation pathway, from hydroxylated intermediates through catechol cleavage products to aliphatic acids, providing direct molecular evidence that the encoded catabolic machinery was operational, not merely present.

**Figure 10:**
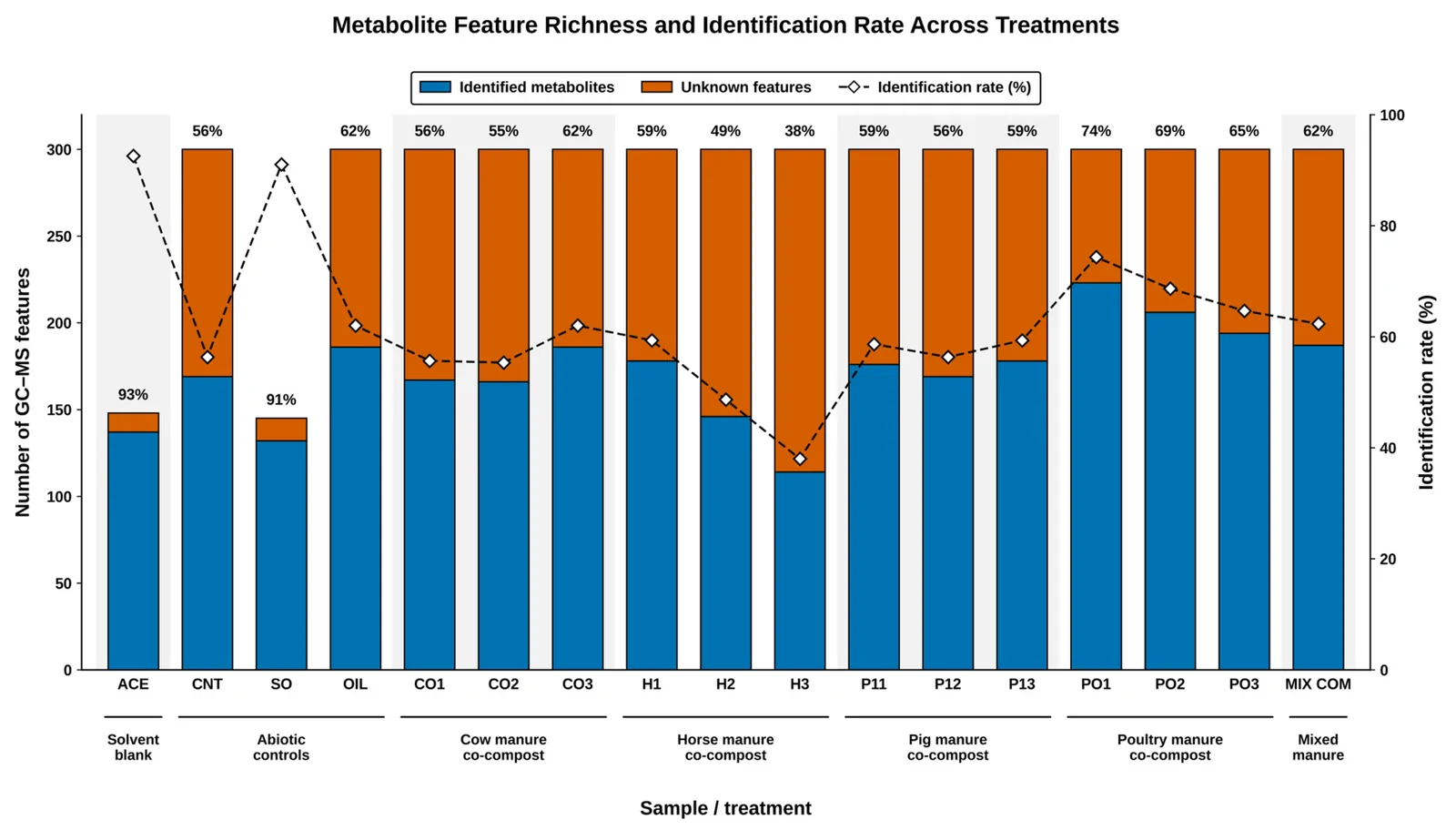
Metabolite feature richness and annotation rate across treatments. Stacked bar charts show annotated metabolites (blue) and unannotated GC-MS features (vermillion) in each sample. The dashed line (right axis) indicates the annotation rate. Bold percentages represent the proportion of annotated features per sample. Codes: ACE, acetone solvent blank; CNT, unamended control; SO, soil-only control; OIL, crude-oil sludge; CO1–CO3, cow; H1–H3, horse; P11–P13, pig; PO1–PO3, poultry; MIX COM, mixed-manure co-compost.

**Figure 11:**
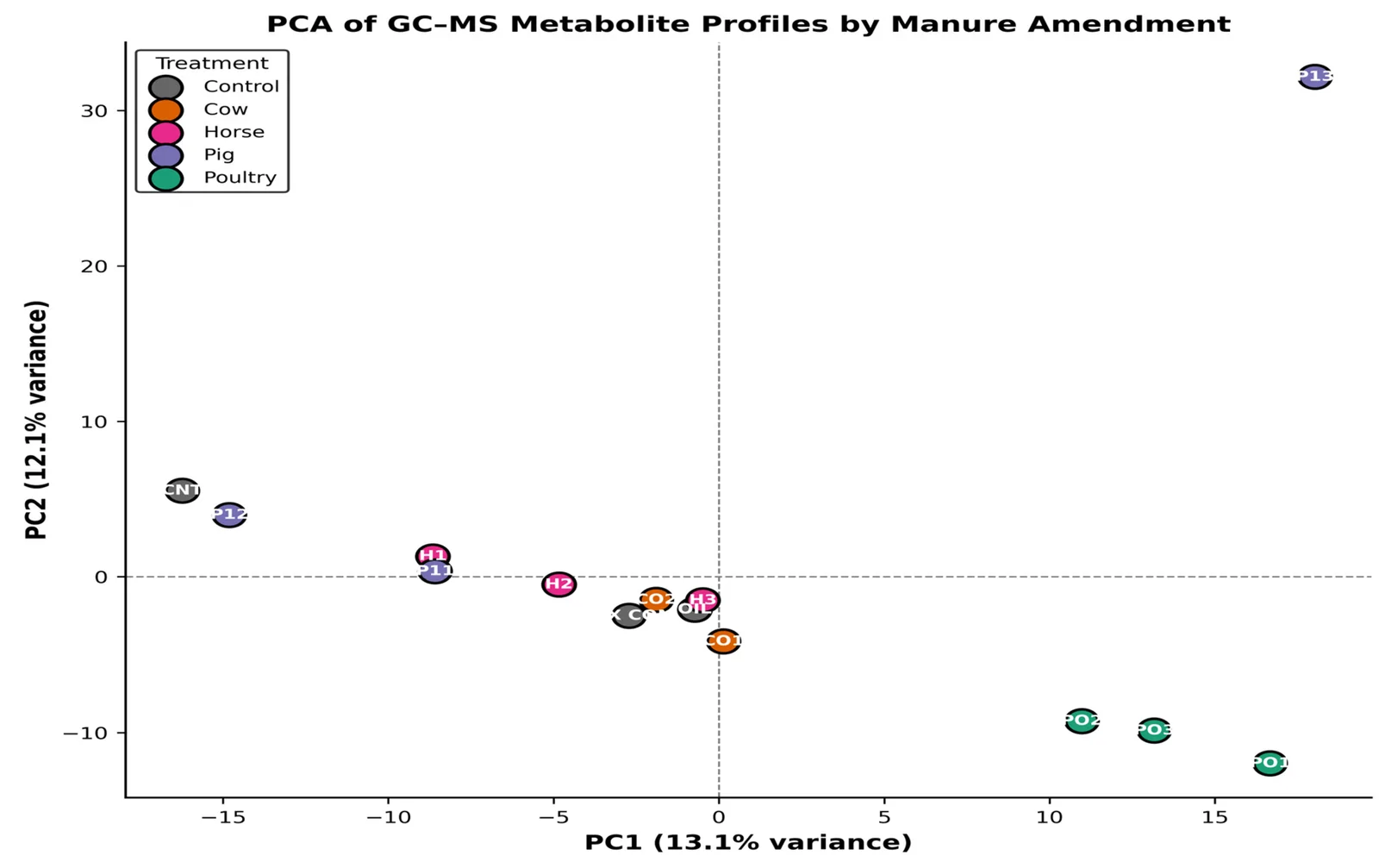
Principal component analysis of metabolite profiles. PCA scores on log-transformed, auto-scaled peak areas; points are coloured by manure type: Replicates cluster by amendment rather than scattering randomly, demonstrating that manure identity reproducibly shapes the metabolite fingerprint. The separation of poultry and pig groups from the control along PC1/PC2 supports the chemical and microbial divergence observed in later figures.

Collectively, the 1169 resolved features provided a molecular atlas of aerobic aromatic catabolism, capturing every successive degradation stage simultaneously (Figure 9). Parent PAHs co-occurred with their hydroxylated intermediates, 2-hydroxyfluorene and phenanthrene-methanol, alongside catechol-type ring-cleavage products such as 1,4-naphthoquinone and benzenediols, and ultimately with the short-chain aliphatic acids (butanedioic, propanoic, and oxalic acids) that signal entry into central carbon metabolism. Conservative petroleum biomarkers, hopane and sterane, persisted throughout as invariant internal reference points against which the depletion of labile constituents could be tracked. Profile richness and annotation depth varied systematically by amendment type. (Figure 10), Oil- and manure-amended microcosms saturated the 300-feature detection window, against 145–148 features in the clean controls (ACE 92.6%, SO 91.0%), quantifying the molecular complexity introduced by the sludge. Poultry replicates achieved the deepest annotation (PO1–PO3, 64.7–74.3%); horse replicate H3 retained the largest unidentified fraction (62.0%), marking amendment-specific transformation products beyond current spectral libraries, and principal component analysis showed that this variation was non-stochastic: replicates clustered tightly by manure type (Figure 11).

### 3.6. Structural Classification and Distribution of Metabolite Chemical Classes

The annotated metabolites were analytically categorized into eleven major chemical classes according to their structural features and biochemical roles, particularly in relation to petroleum biodegradation processes (Table 8). This classification framework highlights the chemical diversity of the detected compounds and provides insight into their functional roles in hydrocarbon-degrading systems. Linking structural characterization with biodegradation potential shows that compound distribution is associated with key metabolic pathways and transformation processes. These processes promote microbial adaptation and enhance degradation efficiency in petroleum-contaminated environments.

### 3.7. Petrogenic Biomarker Fingerprinting and Sequence Biochemical Transformation of PAHs to Ring-Cleavage Intermediates

The untargeted metabolomic analysis showed the petrogenic origin of the crude oil refinery waste sludge by identifying a diagnostic suite of molecular markers across the co-compost treatments. n-Alkanes (C14–C36) consistently displayed an even-over-odd carbon number predominance typical of petroleum hydrocarbons, contrasting with biogenic signatures, while the widespread presence of 1,1,4,5,6-pentamethyl-2,3-dihydro-1H-indene (15/17 samples) showed the persistence of alkylated aromatics. Recalcitrant hopanoid and sterane biomarkers, 17α,21β-28,30-bisnorhopane, androstane, and stigmastane, detected in 8–14 samples served as stable internal references for source origin and biodegradation assessment due to their resistance to microbial alteration [27,28]. The abundant detection of 4-hydroxy-4-methyl-2-pentanone in all samples indicated sustained aerobic microbial activity, while dimethyl diazene (16/17 samples) reflected active nitrogen cycling within the system.

**Figure 12:**
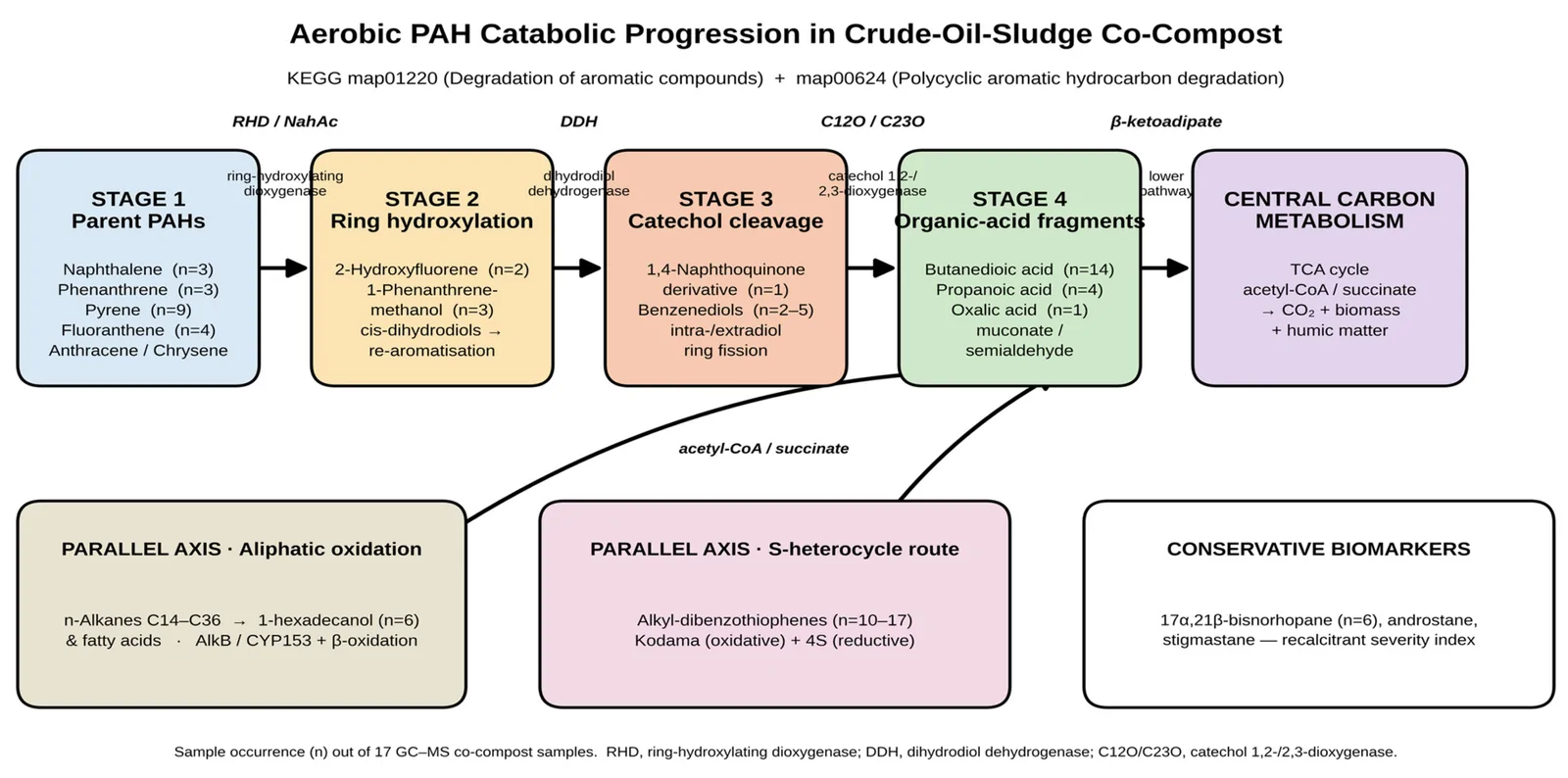
KEGG-anchored biochemical pathway progression reconstructed for crude oil refinery waste-sludge PAH degradation during co-composting. Four sequential stages, namely, parent PAH activation, ring hydroxylation, ring-cleavage (catechol) chemistry, and organic-acid fragmentation, converge on central carbon metabolism (TCA cycle).

Beyond the source origin, the metabolomic profile revealed a coherent, stepwise degradation trajectory of polycyclic aromatic hydrocarbons (PAHs), aligned with the identified aerobic pathways (KEGG map01220; map00624). This progression formed a four-stage cascade supported by experimentally detected intermediates (Figure 12). Diagnostic enzymes are annotated at each transition (ring-hydroxylating dioxygenase, RHD/NahAc; cis-dihydrodiol dehydrogenase, DDH; catechol 1,2- and 2,3-dioxygenase, C12O/C23O; β-ketoadipate route). Parallel auxiliary axes show the aliphatic (AlkB/CYP153), sulfur-heterocycle (Kodama oxidative and 4S/Dsz desulfurisation), and conservative-biomarker channels. The process is mapped to KEGG map01220 (degradation of aromatic compounds) and map00624 (PAH degradation); numbers in parentheses are the count of co-compost samples (17) in which each diagnostic feature was detected.

Parent PAHs, including pyrene, fluoranthene, phenanthrene, and naphthalene, were initially transformed through dioxygenase-mediated hydroxylation. This process generated intermediates, such as phenanthrene-methanol and 2-hydroxyfluorene. The intermediates were subsequently processed through ring-cleavage pathways, as indicated by the detection of benzenediols and 1,4-naphthoquinone. Further transformation yielded low-molecular-weight organic acids, including succinate, propanoic acid derivatives, and oxalic acid esters. These metabolites are consistent with the formation of compounds associated with central carbon metabolism.

**Figure 13:**
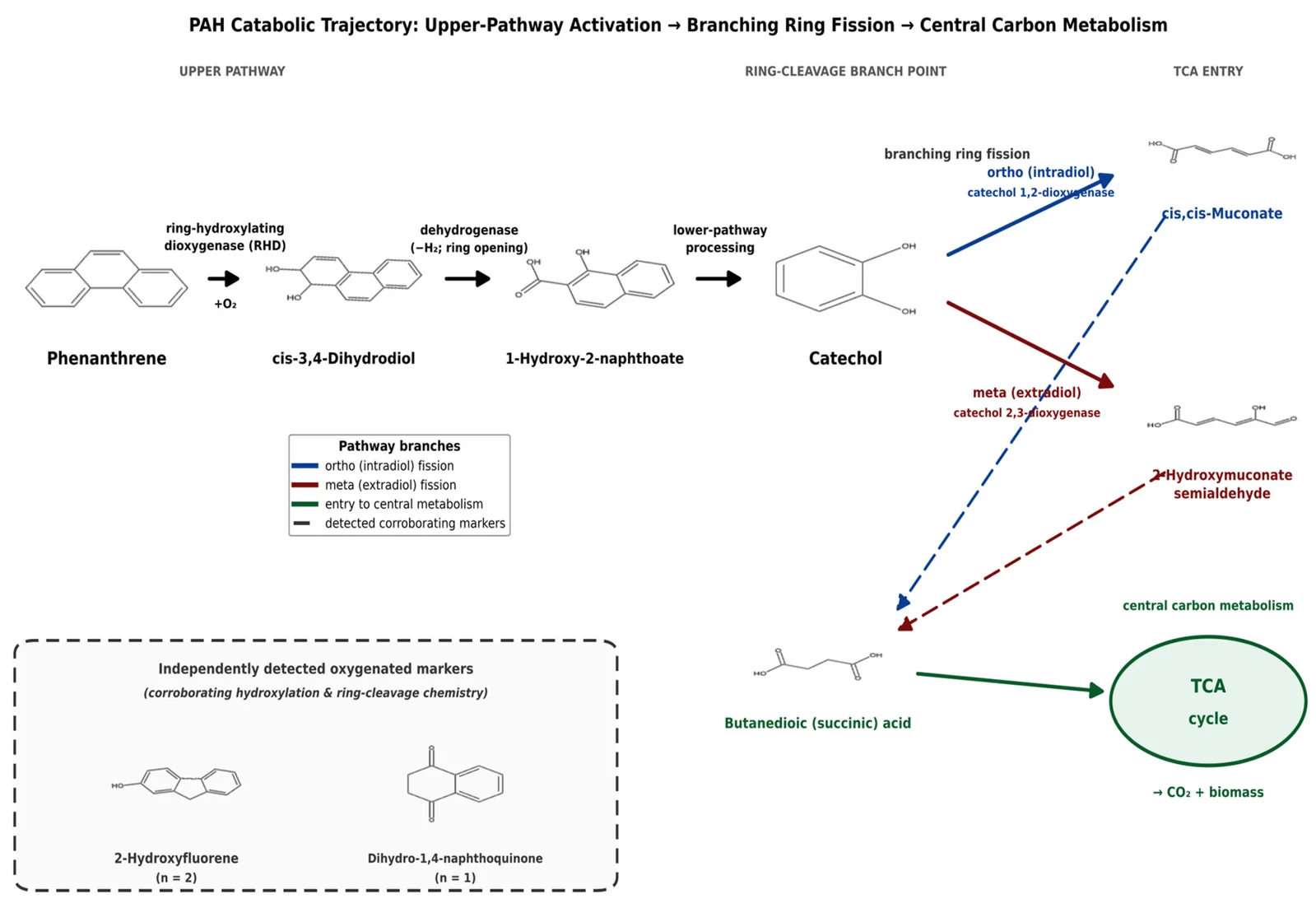
Chemical reconstruction of the putative PAH degradation pathway. Two-dimensional molecular structures illustrating the proposed transformation of phenanthrene through intermediate compounds and ring-cleavage products. The pathway includes ortho- and meta-cleavage branches and the formation of short-chain organic acids associated with central carbon metabolism. The inset shows oxygenated intermediates detected during GC-MS analysis. Structures were rendered using RDKit.

In parallel, aliphatic hydrocarbons appeared to undergo oxidation to fatty alcohols and fatty acids. Detected products included 1-hexadecanol and Z-11-hexadecenal. These compounds are consistent with intermediates that may enter β-oxidation pathways. Together, the detected metabolites suggest concurrent aromatic and aliphatic hydrocarbon transformation processes. Sulfur-containing heterocyclic compounds, including dimethyl-dibenzothiophene isomers, persisted across treatments (Figure 13).

Phenanthrene was predicted to undergo conversion to a cis-3,4-dihydrodiol intermediate, followed by transformation to 1-hydroxy-2-naphthoate and catechol. Subsequent ring cleavage may proceed through either ortho- or meta-cleavage pathways, producing cis,cis-muconate or 2-hydroxymuconate semialdehyde, respectively. These pathways are predicted to generate short-chain organic acids, including succinic acid. Conserved hydrocarbon biomarkers provide a reference for assessing transformation patterns. The detection of pathway-associated intermediates further support the proposed degradation framework. Collectively, these observations suggest the presence of a multi-pathway biodegradation system with the potential to transform complex petroleum hydrocarbons into central metabolic intermediates.

### 3.8. Sulfur-Heteroatom PAHs: Dibenzothiophene Degradation Axis

**Figure 14:**
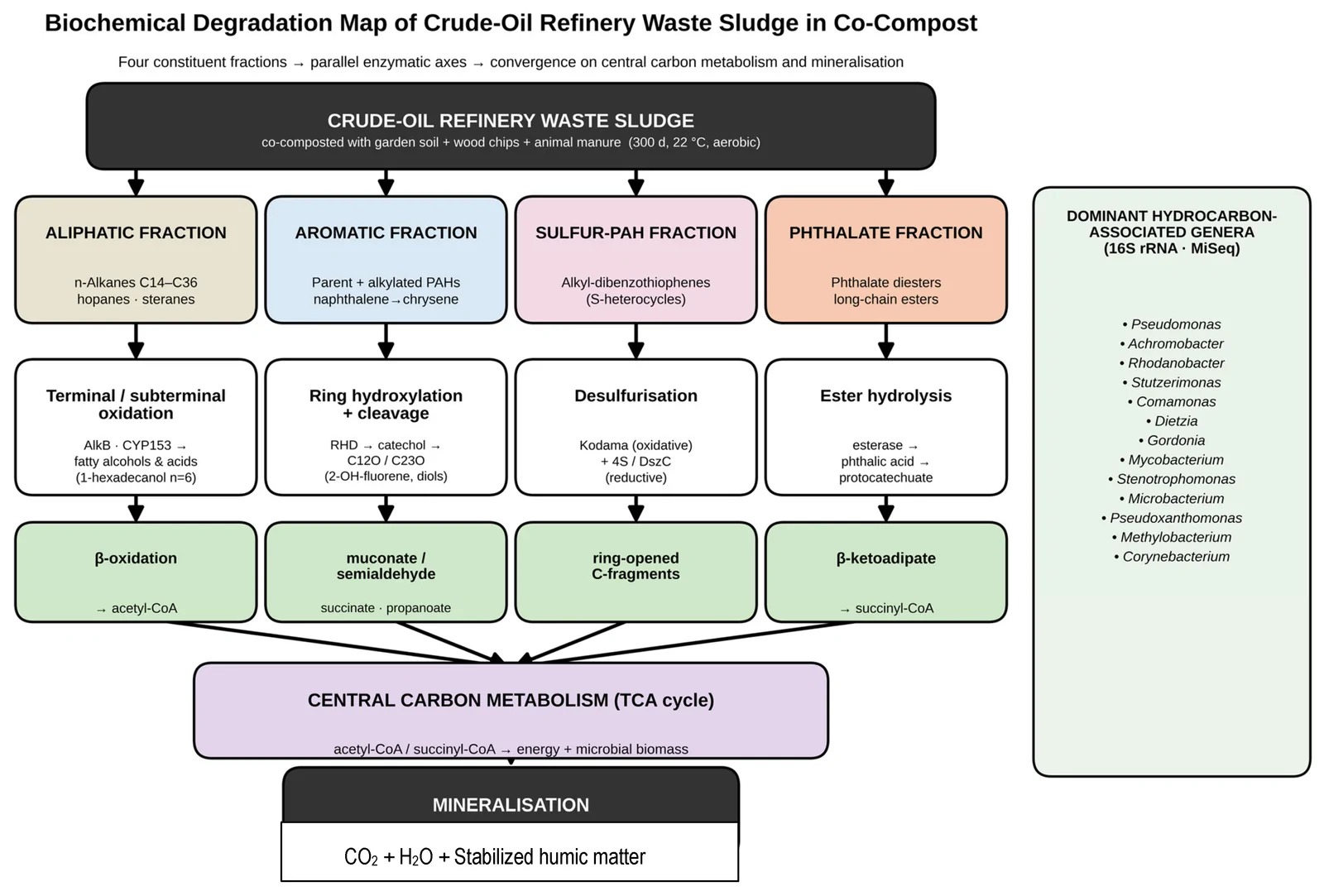
Whole-sludge degradation map. Schematic overview of the proposed transformation pathways for major crude oil refinery sludge fractions, including aliphatic hydrocarbons, aromatic PAHs, sulfur-containing heterocycles, and phthalate esters. The diagram illustrates associated transformation routes, intermediate pools, and links to central carbon metabolism. Dominant hydrocarbon-associated genera identified by 16S rRNA gene sequencing are shown alongside the pathway framework.

Metabolomic profiling revealed a pronounced enrichment of sulfur-heterocyclic PAHs, with the dibenzothiophene (DBT) series constituting a dominant and persistently detected fraction across the crude oil sludge samples. Alkylated DBT homologues, including 1,7- and 2,7-dimethyldibenzothiophene and higher derivatives, were consistently identified in 10–15 samples, predicting their greater resistance to biodegradation relative to carbocyclic PAHs [24,67]. Benzo[b]naphtho [2,3-d]thiophene, 9,10-dihydro-7-methyl- was detected in 15 of the 17 samples, highlighting the persistence of sulfur-containing petrogenic markers. The presence of partially hydrogenated intermediates further suggested the concurrent operation of reductive and oxidative transformation pathways. This observation is consistent with the Kodama and 4S desulfurization mechanisms [68]. Integrated metabolomic network analysis showed that distinct hydrocarbon fractions, including aliphatic, aromatic, sulfur-heterocyclic, and phthalate ester compounds, were degraded simultaneously through pathway-specific enzymatic processes. These pathways converged on a common pool of ring-cleavage intermediates and low-molecular-weight organic acids, which subsequently entered central carbon metabolism and supported mineralization. This coordinated transformation was substantiated by the detection of key metabolites, including 1-hexadecanol (6 samples), alkylated aromatic compounds such as 1,1,4,5,6-pentamethyl-2,3-dihydro-1H-indene (15 samples) and 1,3-dimethylpyrene (14 samples), dimethyl-dibenzothiophenes (9–11 samples), and phthalate esters. Microbial community analysis further reinforced this mechanistic framework, as the dominant genera identified, *Pseudomonas*, *Achromobacter*, *Stutzerimonas*, *Comamonas*, *Dietzia*, *Gordonia*, *Mycobacterium*, and *Rhodanobacter*, collectively possess well-characterized genetic capacities for alkane, aromatic, and sulfur-compound degradation (Figure 14).

The persistence of alkylated and heterocyclic residues likely reflects their reduced susceptibility to degradation, making them important contributors to final compost quality. Furthermore, the co-occurrence of sulfur-containing aromatics and oxygenated intermediates suggests the potential for concurrent desulfurisation, heterocyclic-ring transformation, and carbocyclic PAH degradation, consistent with a diverse repertoire of predicted hydrocarbon-transformation pathways.

### 3.9. Phthalate Ester Dynamics

Phthalate esters, including diisooctyl phthalate, dibutyl phthalate, and bis (2 ethylhexyl) phthalate, were detected in 5–10 samples, indicating their consistent presence across the system. These compounds likely originate from two concurrent sources: (i) external contamination associated with polyvinyl chloride (PVC) composting troughs or plastic constituents embedded within the crude oil sludge matrix and (ii) authentic biodegradation intermediates formed through microbial hydrolysis and aromatic ring-cleavage pathways (KEGG map00624) [69,70]. The co-occurrence of parent phthalate esters with 1,2 benzenedicarboxylic acid derivatives (phthalic acid) provides strong evidence of esterase-mediated diester hydrolysis. This transformation generates phthalic acid as a key metabolic intermediate, which is subsequently funnelled into protocatechuate formation and further metabolised via the β ketoadipate pathway (Figure 14). These findings demonstrate active phthalate biotransformation within the system while also highlighting the potential contribution of background contamination to the detected profiles [69,70].

### 3.10. Unknown Recurring Features: Candidate Novel Biodegradation Products

Among the 631 unidentified features, a distinct subset of 12 unknown compounds (Unknown 1–11 and Unknown 5–9) consistently appeared across all 17 samples, representing the most prevalent unresolved metabolites in the dataset. Additionally, over 30 unknown features were detected in 15–16 samples, indicating a broad pattern of persistence (Table 9). These recurring compounds likely reflect a combination of (i) previously uncharacterized biodegradation intermediates absent from existing spectral libraries, (ii) structurally modified derivatives of known compounds with limited database representation, and (iii) background constituents originating from the sample matrix. Their uniform presence across all treatments, including non-manured controls, suggests that these features either correspond to persistent petrogenic residues or represent abundant metabolites produced by aerobic microbial communities, irrespective of amendment conditions. These findings revealed potential novel transformation products while highlighting the limitations within the current metabolite identification frameworks. The uncharacterized metabolite features identified here therefore warrant systematic investigation to inform the future design of targeted bioremediation strategies.

### 3.11. Amendment-Specific Metabolomic Signatures and GC-MS Identification of Degradation Intermediates

Comparative metabolomic profiling across manure amendment types revealed pronounced differences in both metabolite richness and compositional character, demonstrating that the identity of the organic amendment and, by extension, the microbial community it recruits, fundamentally govern the biochemical trajectory of crude oil sludge degradation. Poultry manure treatments (PO1–PO3) achieved the highest average identification rate (69.2 ± 4.8%), yielding the richest suite of oxygenated intermediates and aromatic degradation products among all amendments. Notably, the exclusive detection of (2-methyl-3-biphenylyl)methanol in PO1 and PO3 indicated that poultry-manure-enriched microbial communities that possessed an enhanced biphenyl hydroxylation capacity were absent from all other treatment groups. In contrast, horse manure treatments (H1–H3) recorded the lowest average identification rate (48.7 ± 10.7%), with replicate H3 annotating only 38.0% of its detected features, a disproportionately high unknown fraction that likely reflects the production of novel or structurally unusual transformation products by functionally distinct microbial consortia derived from equine gut microbiota. Pig manure treatments (P11–P13) occupied an intermediate position (56.3–59.3%), distinguished by a unique enrichment in methylphenol-derived ether metabolites, specifically (3-methylphenyl)methanol 2-methylbutyl ether and (4-methylphenyl)methanol neopentyl ether, detected exclusively in P13, implicating amendment-specific fungal or bacterial methylation–etherification pathways not activated by other manure types. Cow manure treatments (CO1–CO2) maintained consistent identification rates (55.3–55.7%), with the unique detection of (3,4,5,6-tetrahydro-2H-[2,3′]bipyridinyl-1-yl)acetic acid hydrazide in CO2 implicating nitrogen-heterocyclic metabolism as a biochemical signature specific to cow-manure-enriched communities, while the mixed manure co-compost (MIX COM) returned a predictably intermediate identification rate of 62.3%, consistent with a functional averaging of diverse microbial contributions from its constituent amendment sources.

**Figure 15:**
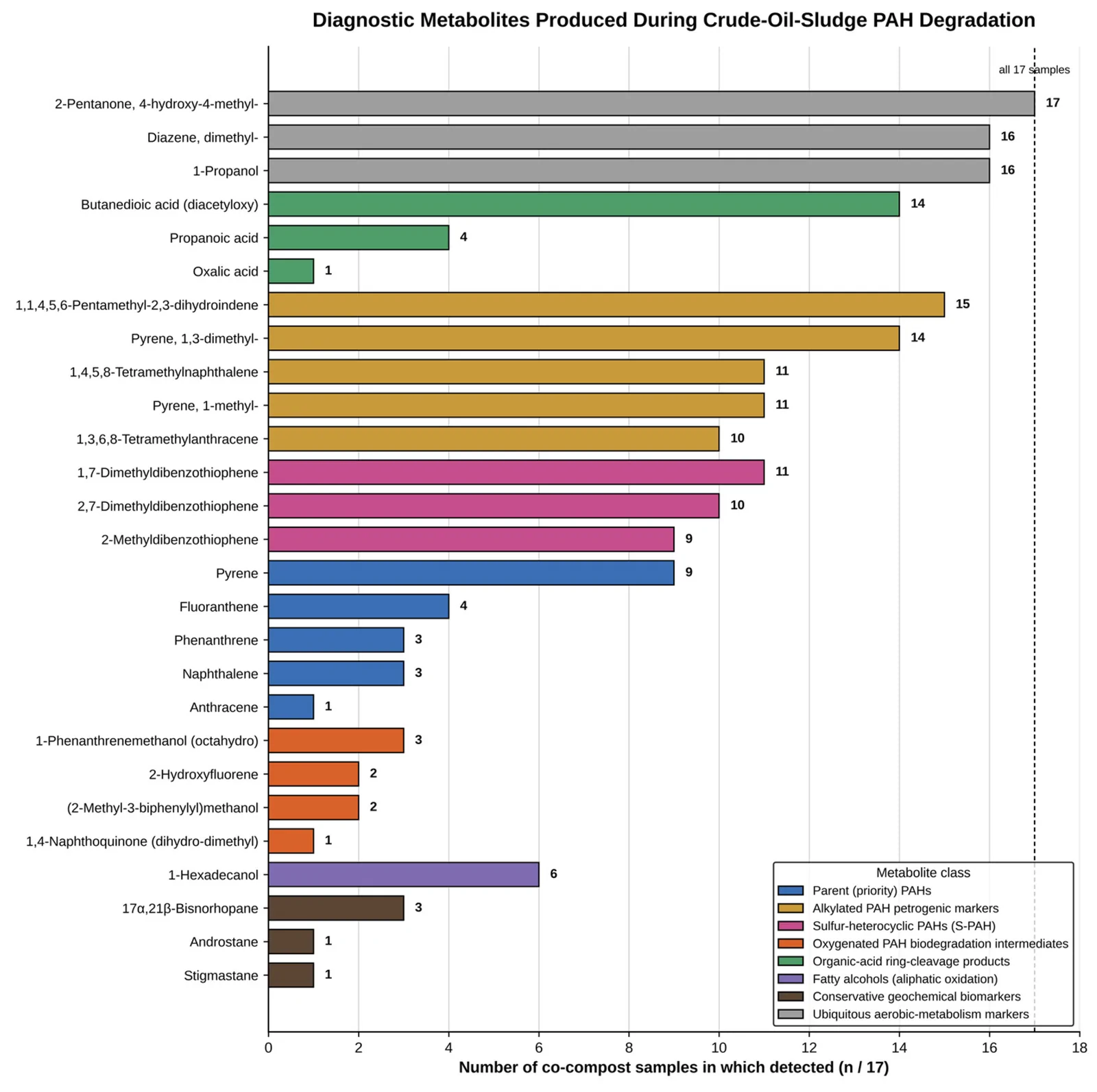
Occurrence frequency of diagnostic metabolites produced during co-composting of crude-oil refinery waste-sludge PAHs expressed as the number of treatments (17) in which each feature was detected. Features are grouped and colour-coded by chemical class (parent PAHs, alkylated PAHs, sulfur-heterocyclic PAHs, oxygenated ring intermediates, organic acids, fatty alcohols, phthalate esters, and conservative biomarkers). The dashed reference line marks ubiquity (detection in all 17 treatments).

Quantifying the metabolite landscape by cross-sample reproducibility revealed an internally coherent mass-balance narrative (Figure 13). Alkylated PAHs and downstream organic acids dominated the high-frequency end of the distribution, including 1,1,4,5,6-pentamethyl-2,3-dihydro-1H-indene (15 of 17 samples), 1,3-dimethylpyrene and the butanedioic acid derivative (14 each), and the dimethyl-dibenzothiophene isomers (10–11 samples), whereas parent PAHs and oxygenated ring intermediates clustered at the low-frequency end, including pyrene (9 samples), fluoranthene (4), phenanthrene and naphthalene (3 each), and hydroxy- and quinone-substituted PAHs (1–3 samples). This frequency gradient reflects active metabolic consumption rather than random variation: parent compounds appear scarce because they are actively degraded, transient oxygenated intermediates remain rare because they turn over rapidly, and short-chain organic acids accumulate at high frequency as the convergent terminal products of multiple upstream degradation routes. The colour-coded chemical class assignments additionally reflect relative compound recalcitrance. The persistence of alkylated and sulfur-substituted aromatics at high frequencies suggests that these hydrocarbon fractions are more resistant to attenuation, whereas the reduced abundance of unsubstituted parent aromatic compounds may indicate their greater susceptibility to microbial transformation. Conservative biomarkers, 17α,21β-bisnorhopane and steranes (1–3 samples), functioned as intended non-degradable internal references, and the fatty alcohol 1-hexadecanol (6 samples) marked the active aliphatic oxidation channel, together communicating not merely which metabolites form but also what their relative detection frequencies reveal about metabolic flux and rate-limiting blockages (Figure 15).

Table 10 summarizes each identified metabolite, including its molecular formula, GC retention time, quantifier ion, assigned pathway role, and Metabolomics Standards Initiative (MSI) confidence level. The entries span the complete degradation cascade, encompassing oxygenated ring intermediates (2-hydroxyfluorene, octahydrophenanthrenemethanol, biphenylylmethanol, and dihydro-1,4-naphthoquinone), short-chain organic acids (butanedioic acid derivative, propanoic acid, and oxalic acid), the fatty alcohol 1-hexadecanol, dimethyl-dibenzothiophene isomers, phthalate esters, and 17α,21β-bisnorhopane as the conservative petroleum biomarker. The NIST spectral similarity indices, consistently above 940 for well-resolved acids and alcohols and ranging from 660 to 820 for trace oxygenated PAHs, provided a transparent, per-feature measure of identification certainty rather than an undifferentiated compound list. The explicit reporting of MSI confidence levels distinguishes metabolites identified with high confidence from those assigned provisionally, allowing the strength of the supporting evidence to be evaluated transparently. High-confidence frequently detected organic acids provide stronger support for the proposed transformation pathways, whereas lower-confidence oxygenated PAHs should be regarded as candidate intermediates requiring further verification. This framework enables the interpretation of metabolomic data within clearly defined confidence boundaries.

### 3.12. Endogenous Biosurfactant Production and Surface Activity Dynamics of the Microbial Consortium: Implications for Hydrocarbon Bioavailability Enhancement

To assess whether the consortium could endogenously produce biosurfactants capable of enhancing hydrocarbon bioavailability and reducing reliance on exogenous Tween 80, all 27 active bacterial isolates were screened and pooled in equal volumes (1 mL per isolate; 1:1, *v*/*v*) to form a single consortium (Table 11). The twenty-seven isolates were biosurfactant producers, representing all source categories: cow, horse, pig, and poultry manure composts; mixed compost; the control; and crude oil sludge. This broad distribution indicates substantial surface-active potential across the consortium.

**Figure 16:**
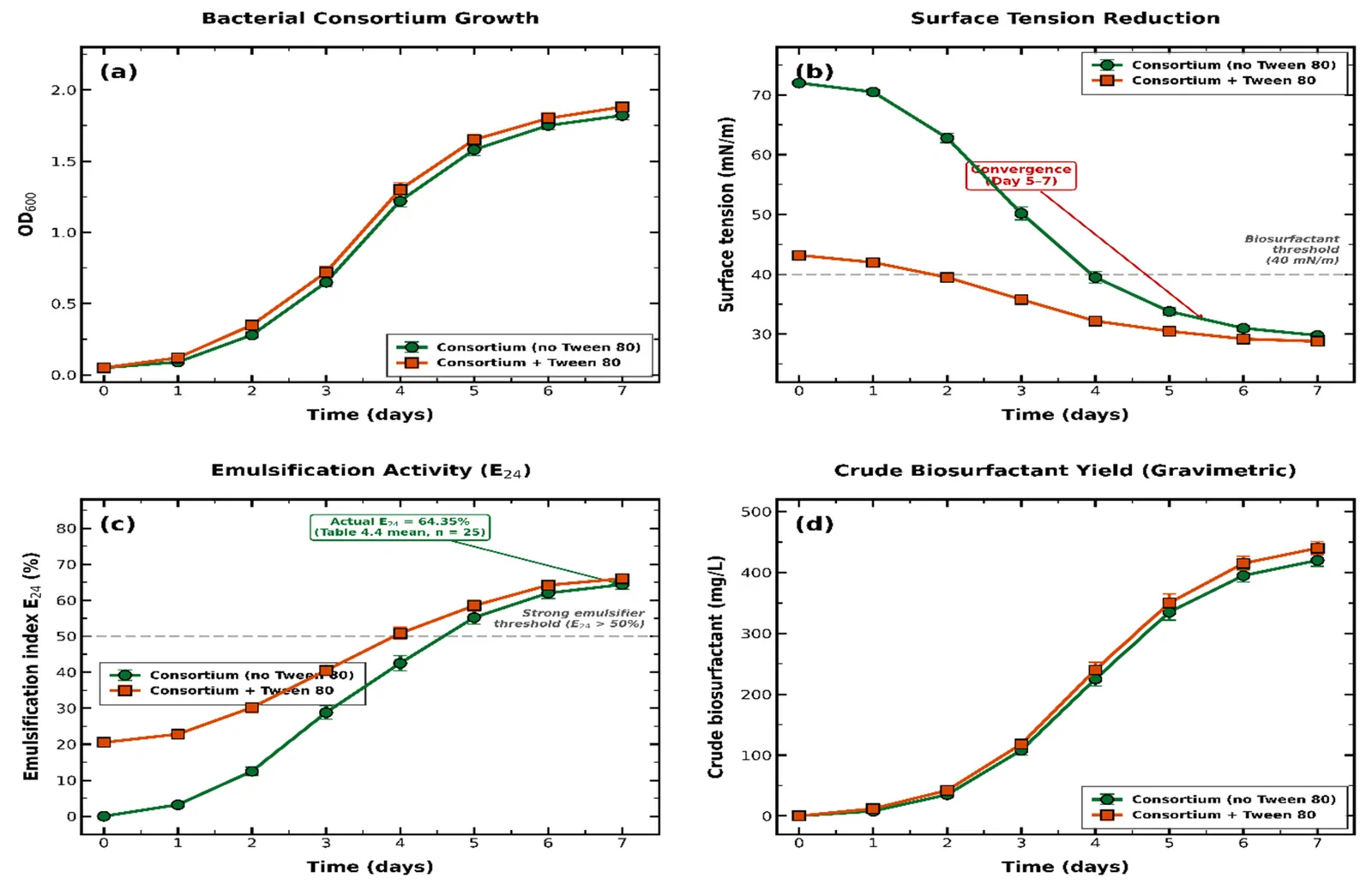
Time-course profiles of bacterial growth and biosurfactant-related parameters. (**a**) Bacterial consortium growth (OD_600_), (**b**) surface tension of cell-free supernatants, (**c**) emulsification index (E_24_), and (**d**) crude biosurfactant concentration measured over 7 days in cultures with and without Tween 80. Cultures were incubated at 30 °C and 150 rpm. Cell-free supernatants were obtained by centrifugation at 8500× *g* for 10 min at 4 °C. Values represent mean ± SD.

A comprehensive seven-day time-course experimental design was implemented, incorporating surface tension, emulsification index (E_24_), crude biosurfactant yield, oil displacement, and biomass accumulation measurements on cell-free supernatants collected at 24-h intervals (Table 12 and Figure 16a–d).

In unsupplemented cultures, surface tension declined progressively from 72.00 ± 0.26 mN m^−1^ at Day 0 to 29.80 ± 0.37 mN m^−1^ by Day 7, a 42.2 mN m^−1^ (58.6%) reduction, crossing the widely accepted biosurfactant-indicative threshold of 40 mN m^−1^ by Day 4, comparable to values reported for established biosurfactant-producing strains (Figure 16b). Concurrently, emulsification capacity rose from 0% at Day 0 to 64.35 ± 1.32% by Day 7, surpassing the strong emulsifier threshold of 50% by Day 5. Meanwhile oil displacement assays yielded a mean clearing zone of 2.09 ± 0.56 cm, approximately 75% of the Tween 80 control, and drop-collapse scores progressed systematically from a beaded negative response at Day 0 to complete collapse by Day 6 (Figure 16c). Crude biosurfactant yield climbed from undetectable levels to 420 ± 9.5 mg L^−1^ by Day 7 (Figure 16d), and the synchronous directional movement of all five independent readouts rendered the signal internally supported and robust, providing direct quantitative evidence that the consortium actively synthesizes a functional biosurfactant during sludge degradation rather than merely harbouring genera with theoretical biosurfactant capacity. At each time point, the pairwise comparison is consortium vs. consortium + Tween 80. Based on the convergence in the data pattern annotated, the following was observed: from Day 0 to Day 3, the conditions were clearly different (***; *p* < 0.001); on Day 4, the gap was closing (**; *p* < 0.01); and from Day 5 to Day 7, convergence was achieved (ns; *p* > 0.05) (values = mean ± SD; Day-7 E24: consortium mean of 25 positive producers = 64.35 ± 1.32%).

The taxonomic and genetic basis of this surface activity was elucidated by pairing the 16S rRNA community census with literature-documented biosurfactant gene repertoires for each recovered genus. *Pseudomonas* and *Stutzerimonas* produce rhamnolipid-type glycolipids encoded by *rhlA*, *rhlB*, *and rhlC*; the actinobacterial genera *Dietzia*, *Gordonia*, *Mycobacterium*, and *Corynebacterium* synthesize trehalose-lipid and mycolate glycolipids via *treS/treY/treZ* and *mmpL3*; *Stenotrophomonas* contribute lipopeptides through NRPS-encoded *ituA–D* gene clusters; and *Comamonas* produces polymeric bioemulsifiers via *emcA/emcB* [71,72,73,74,75,76]. The observed association between community composition, predicted biosurfactant production potential, and metabolite profiles suggests that 16S rRNA-derived community structure may indicate functional traits relevant to hydrocarbon transformation. The dominance of putative glycolipid-producing taxa corresponded with the low supernatant surface tension (~29 mN m^−1^) observed in these systems, consistent with a potential role in hydrocarbon desorption and solubilisation prior to subsequent microbial degradation (Figure 14) [77,78,79,80,81,82,83,84,85].

Comparative analysis of Tween-80-supplemented and unsupplemented cultures revealed that, although exogenous surfactant conferred an early bioavailability advantage, holding surface tension at 43.2 mN m^−1^ at Day 0 and supporting 20.5% emulsification and a 1.77 cm^2^ displacement zone through the first three days, both treatment regimes converged from Day 5 onward, with no statistically significant differences in surface tension or E_24_ values (*p* > 0.05; two-way ANOVA with Tukey’s HSD; Figure 16b,c). Final Day 7 surface tensions (29.80 ± 0.37 and 28.80 ± 0.26 mN m^−1^), E_24_ values (64.35 ± 1.32% and 66.00 ± 1.16%), and biosurfactant yields (420 ± 9.5 and 440 ± 10.6 mg L^−1^) were comparable between the unsupplemented and Tween-80-amended cultures, indicating minimal differences in biosurfactant production and emulsification performance [86,87,88]. Collectively, these findings suggest that COWS degradation in animal-manure-amended compost is associated with a functionally diverse microbial community and its predicted metabolic potential. Endogenously produced biosurfactants achieved surface activity comparable to that observed with Tween 80 supplementation, indicating that Tween 80 may enhance hydrocarbon bioavailability during the early stages of composting but has limited influence once microbial biosurfactant production becomes established. Overall, the results are consistent with multiple, potentially complementary hydrocarbon-transformation pathways. The results reveal consistent associations among amendment composition, microbial community dynamics, respiratory activity, PAH attenuation, and the accumulation of transformation intermediates. Integrating these complementary datasets provides a comprehensive framework for understanding the microbial and biochemical processes potentially involved in hydrocarbon transformation during composting.

## 4. Discussion

This study advances petroleum bioremediation research by integrating untargeted GC-MS metabolomics, targeted PAH analysis, 16S rRNA community profiling, and physicochemical monitoring within a single co-composting experiment. Unlike studies that primarily focus on bulk degradation endpoints, this approach provides a more detailed assessment of microbial, biochemical, and predicted functional changes associated with hydrocarbon transformation. Previous studies have often relied on total petroleum hydrocarbon or total PAH removal as the primary measure of treatment performance, providing limited insight into transformation pathways and the microbial communities involved [14].

A total of 1169 metabolite features were detected and linked to KEGG degradation pathways (map01220 and map00624) [60,61]. Microbial community composition was assessed in parallel. Together, these datasets enabled direct comparison of metabolite profiles, taxonomic shifts, and predicted functional potential.

Manure-amended treatments achieved higher PAH removal than the unamended control. Mean PAH removal ranged from 79% to 88% in manure-treated composts, compared with 68.2% in the control (Figure 6 and Table 6). The experimental design minimised nutrient-related variability. The sandy loam substrate provided a relatively low baseline degradative capacity (Table 2), whereas the four manure amendments differed in carbon and phosphorus content but had comparable nitrogen concentrations (Table 3).

Low-molecular-weight PAHs, particularly two- and three-ring compounds, showed the highest removal efficiencies and frequently exceeded 99%. This pattern is consistent with the greater bioavailability of smaller PAHs reported in previous studies [17,89]. Ash content remained relatively stable throughout the composting period (Table 4), indicating that the mineral fraction of the matrix was largely unchanged. This observation suggests that reductions in contaminant concentrations were primarily associated with the loss of organic material rather than redistribution within the compost matrix.

At the microbial level, 16S rRNA sequencing identified 2969 OTUs distributed across 15 phyla and 288 genera (Figure 2 and Figure 3). Community composition matched patterns commonly reported in hydrocarbon-contaminated environments. *Pseudomonas* were particularly abundant in the highest-performing treatments. In contrast, cow manure treatments showed greater representation of the actinobacterial genera *Dietzia*, *Gordonia* and *Mycobacterium*.

The metabolite–microbe interaction network (Figure 4) highlighted *Pseudoxanthomonas*, *Stutzerimonas*, and *Achromobacter* as key taxa associated with metabolite transformation patterns. Alpha-diversity analysis revealed distinct community assembly strategies (Figure 5 and Table 5). Pig manure treatments combined high diversity and evenness (Shannon = 4.27; Simpson = 0.975) with strong PAH removal. Horse manure treatments achieved comparable removal despite lower community evenness and greater dominance by fewer taxa.

These results suggest that treatment performance was associated with community structure rather than amendment composition alone. Metabolites associated with ortho- and meta-cleavage pathways were detected and aligned with reported aromatic hydrocarbon degradation routes [67]. Physicochemical measurements further indicated active composting conditions (Figure 1). Poultry manure treatments yielded the highest respiration rates, reaching 18.7 mg CO_2_-C g^−1^. Compost pH temporarily increased towards neutrality (7.8–7.9), and temperatures remained within the mesophilic range throughout the study. Together, these observations indicate conditions conducive to microbial activity and hydrocarbon transformation.

Framing these results within a KEGG-based framework places them in the context of established models of bacterial aromatic hydrocarbon degradation [67,90,91]. In these pathways, upper-pathway reactions convert diverse PAHs into a smaller set of oxygenated intermediates. Lower-pathway processes subsequently transform these compounds into metabolites associated with central carbon metabolism.

The metabolite dataset included compounds from eleven chemical classes (Table 8) and represented multiple stages of the proposed degradation process (Figure 9 and Figure 12). Detected compounds included parent PAHs, hydroxylated intermediates such as 2-hydroxyfluorene and phenanthrene-methanol, ring-cleavage products including benzenediols and 1,4-naphthoquinone, and short-chain organic acids such as butanedioic, propanoic, and oxalic acids. Together, these metabolites are consistent with sequential hydrocarbon transformation.

The pathway reconstruction shown in Figure 13 illustrates a putative progression from cis-dihydrodiols to catechol intermediates, followed by ring cleavage through ortho- or meta-cleavage pathways [67]. These routes channel carbon through the β-ketoadipate or hydroxymuconate semialdehyde pathways, respectively.

A key strength of this study is the integration of metabolomic, taxonomic, and pathway-based analyses [92,93,94,95,96]. The proposed pathway framework was supported by detected metabolites with defined annotation confidence levels (Table 10). NIST similarity indices exceeded 940 for several well-resolved acids and alcohols and ranged from 660 to 820 for oxygenated PAHs present at lower abundances. This approach aligns with metabolomics reporting guidelines that emphasize evidence-based compound annotation and transparent confidence reporting [49].

Several observations differed from the expected patterns and provided important directions for future research. First, organosulfur heterocycles remained detectable throughout the study. Dimethyl-dibenzothiophenes were detected in 9–11 samples, while benzo[b]naphtho [2,3-d]thiophene derivatives occurred in 15 of 17 samples. These compounds represent a hydrocarbon fraction that is often overlooked in composting studies focused primarily on homocyclic PAHs. The detection of partially hydrogenated sulfur-containing compounds suggests the coexistence of multiple desulfurization pathways [68]. This pattern is reflected in the whole-sludge degradation framework proposed in Figure 14.

Second, oxygenated PAHs (OPAHs) have received limited attention in most composting studies despite concerns regarding their toxicity and mobility [24,26]. In this study, compounds such as 1,4-naphthoquinone and related oxygenated intermediates were detected. Their occurrence alongside downstream organic acids suggests continued transformation during composting rather than simple accumulation [24,26,97]. These findings highlight the importance of monitoring transformation products in addition to parent PAHs when evaluating remediation outcomes.

Third, a substantial proportion of the detected features remained unannotated. The highest proportion occurred in horse manure replicate H3, where 62% of features could not be assigned (Figure 10). Many of these unknown compounds were consistently detected across treatments. More than 30 features occurred in 15–16 of the 17 samples (Table 9). Their persistence suggests the presence of previously uncharacterized biodegradation products, structurally modified derivatives with limited spectral library representation, or residual petrogenic compounds.

These unknown features may arise from transformation processes that are not well represented in current databases. Potential contributors include oxidative reactions mediated by microbial enzymes associated with lignocellulose degradation [98,99]. Although their identities remain unresolved, the consistent occurrence of these compounds highlights priorities for future tandem mass spectrometry and structural characterization studies. The findings also illustrate both the value of untargeted metabolomics and the current limitations of reference-based compound annotation.

A key strength of this study is the use of metabolite detection frequency across multiple treatments as the primary analytical framework (Figure 15). This approach differs from studies based on individual microcosms and emphasizes consistently detected features over isolated observations [20,21,23]. Principal component analysis (Figure 11) showed clear clustering by manure type, indicating treatment-specific metabolite profiles.

The decline in parent PAHs coincided with an increase in organic acids. This pattern is consistent with the progressive hydrocarbon transformation and complements the targeted PAH removal data [67,89,91]. The inclusion of 17α,21β-bisnorhopane and steranes as conservative reference compounds provided an additional basis for interpreting changes in more labile hydrocarbon fractions [27,28].

This study enhanced metabolite annotation transparency by assigning Metabolomics Standards Initiative (MSI) confidence levels and reporting NIST similarity indices for all detected features [49], a more rigorous approach than that of many bioremediation studies that report GC-MS identifications without indicating annotation reliability. Among the 20 intermediates detected by GC-MS (Table 10), 5 metabolites achieved MSI Level 1 confidence through verification with authentic standards or high-quality reference spectra. These included the short-chain organic acids (butanedioic acid derivative, propanoic acid, and oxalic acid), 1-hexadecanol, and dibutyl phthalate, all with NIST similarity indices of 943–972. Thirteen metabolites were classified as MSI Level 2 based solely on spectral library matches, whereas a C_27_ sterane (similarity index 660) and nitrogen-containing hydrazide (710) were assigned to MSI Level 3. Consequently, 75% of the annotations relied exclusively on library-based identification, limiting confidence in the structural assignment and interpretation of their proposed biodegradation roles.

Several constraints arise from this reliance on spectral matching. Electron-ionisation mass spectra are often conserved among structurally related aromatic compounds, making it difficult to distinguish positional and substitutional isomers based solely on fragmentation patterns. This challenge is exemplified by the 1,7- and 2,7-dimethyldibenzothiophene isomers, whose spectra provide minimal diagnostic differentiation. Similar uncertainty affects alkylated PAHs because methyl-substitution positions cannot be determined reliably from mass spectral data alone. Confidence among MSI Level 2 annotations also varied considerably (similarity indices 761–910), with octahydrophenanthrenemethanol (761), a benzo[b]naphtho [2,3-d]thiophene derivative (780), (2-methyl-3-biphenylyl)methanol (793), and 2,7-dimethyldibenzothiophene (798) falling below the commonly accepted confidence threshold of 800. Additional sources of uncertainty included co-elution, matrix interference, background-subtraction artefacts, and incomplete representation within spectral libraries, which may contribute to misannotation, particularly for the low-scoring MSI Level 3 compounds. Furthermore, because the dataset is semi-quantitative, occurrence frequencies should be interpreted as measures of relative prevalence rather than as absolute concentrations.

These limitations do not compromise the overall biodegradation pathway reconstruction, which is supported by high-confidence MSI Level 1 metabolites, particularly the organic acids indicative of terminal mineralization. However, assignments involving oxygenated aromatic intermediates and heterocyclic PAHs, which are critical for evaluating residual toxicity and ecological risk, remain largely provisional because they fall within the MSI Level 2 category. Targeted MS/MS fragmentation analyses and retention index verification against authentic standards are therefore recommended to improve annotation confidence and strengthen future ecological and toxicological assessments [49].

The integration of community structure with bioavailability mechanism represents a further advance over much of the prior composting research, which has typically reported either chemistry or community structure but rarely reconciled both within one explanatory framework [14,100,101]. Biosurfactants are central to PAH bioremediation because they raise the aqueous availability of hydrophobic substrates [13]. Our time-resolved assays (Table 11 and Figure 16) document biosurfactant production as a measured phenotype of the crude-oil-sludge-degrading consortium rather than an inferred capacity. The convergence of surface tension and emulsification index between unsupplemented and Tween-80-supplemented cultures from Day 5 onward (*p* > 0.05) reveals a self-regulating bioavailability mechanism. The consortium autonomously synthesised biosurfactants in direct response to the substrate, reducing surface tension by 42.2 mN m^−1^ (from 72.00 to 29.80 mN m^−1^), accumulating 420 ± 9.5 mg L^−1^ of crude biosurfactant by Day 7, and achieving 64.35% emulsification alongside complete drop collapse. This substrate-induced, growth-associated kinetic profile, with peak production during the exponential phase (Days 2–5), mirrors the primary-metabolite behaviour reported for lipopeptide-producing *Bacillus* spp. and rhamnolipid-producing *Pseudomonas* spp. [102,103,104]. Notably, the consortium achieved surface tension reduction exceeding that of established single-strain producers such as *Pseudomonas aeruginosa PG1* (ΔST = 22.2 mN m^−1^) [105], a performance best explained by a biosurfactant cocktail effect arising from taxonomic and functional diversity: rhamnolipid glycolipids from *Pseudomonas* and *Stutzerimonas*; trehalose-lipid and mycolate glycolipids from the actinobacterial *Dietzia*, *Gordonia*, *Mycobacterium*, and *Corynebacterium* [17,18]; lipopeptides from *Stenotrophomonas*; and polymeric bioemulsifiers from *Comamonas*. A terminal surface tension near 29 mN m^−1^ is fully consistent with the performance ceiling of glycolipid surfactants, directly linking the measured function to the producer genera recovered by sequencing [16,106]. This finding challenges the prevailing assumption that exogenous surfactants are required to enhance hydrocarbon bioavailability. The statistical redundancy of Tween 80 at a steady state argues against routine surfactant dosing and instead supports leveraging indigenous producers, thereby reducing the economic and ecotoxicological costs associated with field-scale application [13]. However, its early-phase benefit highlights a practical strategy: applying a small initial surfactant dose to shorten the lag phase until microbial production dominates within a two-phase kinetic framework [107].

Integrating PICRUSt2 functional predictions with amplicon and metabolomic data addresses a key limitation of bioremediation studies: the detection of degrader taxa alone does not demonstrate functional degradation potential. A total of 7160 KEGG Orthologs were recovered across all enzymatic stages of hydrocarbon degradation (Table 7 and Figure 7 and Figure 8). Poultry manure treatments showed enrichment of ortho-cleavage and β-ketoadipate pathway enzymes, including catechol 1,2-dioxygenase (K03381), muconate cycloisomerase (K01856), and 3-oxoadipate enol-lactonase (K01055). In contrast, the actinobacterial cow manure community was enriched in alkane- and benzoate-oxidation genes, including alkB (K00496) and benzoate 1,2-dioxygenase (K03379). These functional differences provide independent support for the treatment-specific metabolite profiles and contaminant removal efficiencies. This three-way alignment, with taxonomy, predicted function, and measured metabolites converging on a single functional narrative, represents a level of evidential integration uncommon in composting-based bioremediation studies. Collectively, these results indicate that crude oil waste sludge degradation in animal-manure-amended compost is driven by a genetically complementary microbial consortium. *Pseudomonas* and *Stutzerimonas* appear to initiate aromatic-ring degradation while enhancing hydrocarbon bioavailability through rhamnolipid production (*rhlA/rhlB/rhlC*). *Mycobacterium* and *Gordonia* contribute to the breakdown of high-molecular-weight PAHs and sulfur-containing heterocycles through pathways associated with *nidA/B*, *dszA-D*, and trehalose-lipid biosynthesis genes. *Achromobacter* and *Comamonas* support biphenyl and phthalate degradation via *bphABCD* and *ophA-C*. In parallel, *Dietzia* and *Rhodanobacter* facilitate long-chain alkane oxidation through *ladA*, *almA*, and *CYP153*. The observed associations among microbial community composition, predicted functional potential, and metabolite profiles suggest the involvement of a diverse microbial assemblage with complementary hydrocarbon-transforming capabilities. Overall, these patterns are consistent with the operation of multiple putative pathways distributed across the community, highlighting the importance of microbial interactions in shaping hydrocarbon transformation processes.

This study provides a cross-disciplinary framework that integrates amplicon-derived taxonomic profiles, metabolomic signatures, and chemical kinetics to enable comparative interpretation across multiple datasets. Treatment-specific patterns suggest functional partitioning among hydrocarbonoclastic genera, indicating that amendment chemistry may influence microbial community assembly and predicted biodegradation potential. The apparent enrichment of degrader-associated taxa under specific manure treatments highlights opportunities for developing targeted bioaugmentation and biostimulation approaches.

The RDKit-derived pathway map provides a reproducible platform for exploring putative enzyme targets and supporting constraint-based metabolic modelling. Linking detected biochemical intermediates with measured hydrocarbon removal trends further establishes a metabolite-informed framework for assessing biodegradation progress and predicting treatment performance. The persistence of alkylated PAHs and dimethyl dibenzothiophenes suggests that alkyl- and sulfur-substituted homologues may represent slower-transforming fractions that contribute to residual toxicity and influence final compost quality. In addition, the concurrent detection of oxygenated PAH (OPAH) intermediates alongside decreases in parent compounds highlights the potential need for process optimization, including extended maturation and curing periods, improved aeration, and biochar supplementation, to reduce transient OPAH accumulation during composting [9,48].

### Limitations and Future Directions

This study used GC-MS metabolomic profiling at a single endpoint (300 days) to evaluate bioaugmentation and biostimulation outcomes across the composting process, including maturation and curing phases. This approach provided insight into cumulative transformation patterns, residual toxicity indicators, and final compost quality. However, endpoint sampling limited assessment of temporal metabolic dynamics and prevented reconstruction of degradation kinetics.

A substantial proportion of metabolite features remained unannotated (54%; Table 9). This finding highlights the need for advanced annotation approaches, including tandem mass spectrometry (MS/MS) and GNPS-based molecular networking. These methods could improve the characterization of recurrent unknown compounds.

Functional profiles were inferred using PICRUSt2. Although supported by low NSTI values (0.05–0.16), PICRUSt2 predicts functional potential from 16S rRNA data rather than direct genomic measurements. As a result, it cannot assess gene expression patterns, enzyme activity, strain-level variation, or the contribution of mobile genetic elements to hydrocarbon degradation.

Statistical analysis should also be considered exploratory. Results were interpreted using both effect sizes and adjusted significance metrics to support biologically relevant inferences.

Future studies should incorporate time-resolved multi-omics approaches, including shotgun metagenomics and metatranscriptomics. These methods would enable direct assessment of gene content and transcriptional activity. Additional work could include quantitative PCR of key catabolic genes (*nahAc*, *catA*, *alkB*, and *dszC*). Absolute metabolite quantification using deuterated internal standards would further strengthen pathway interpretation. LC-MS-based biosurfactant profiling, combined with metatranscriptomic analysis of biosurfactant biosynthesis genes (*rhlA*, *rhlB*, *rhlC*, *rhlR/rhlI*, *treS/treY/treZ*, *mmpL3*, *ituA-D*, *srfAA-AD*, and *emcA/emcB*), could provide a more detailed understanding of biosurfactant production. Together, these approaches would improve the characterization of biosurfactant composition and help resolve the potential functional contributions of individual consortium members.

## 5. Conclusions

This study presents a robust untargeted GC-MS metabolomic characterization of crude oil waste sludge co-composted with cow, horse, pig, and poultry manures over 300 days, resolving 1169 metabolite features, including petrogenic markers, aromatic ring-cleavage intermediates, sulfur-PAH derivatives, and oxygenated by-products. The resulting profiles defined a coherent biochemical continuum from parent hydrocarbons through hydroxylation and catechol cleavage to integration into central carbon metabolism, aligning with established KEGG aromatic degradation pathways. Poultry manure produced the most diverse and advanced metabolite signatures, indicating a strong stimulation of hydrocarbon-degrading communities, whereas horse manure exhibited the highest proportion of unidentified compounds, suggesting the formation of potentially novel transformation products. Nevertheless, PAH mean-removal efficiency followed the order: pig (88.0%) > horse (87.0%) > poultry (80.5%) > cow (79.1%) > control (68.2%). This demonstrated that microbial functional complementarity and community evenness, rather than nutrient content alone, governed degradation performance.

The concurrent decrease in parent hydrocarbons and accumulation of oxygenated intermediates is consistent with ongoing hydrocarbon transformation and highlights the importance of monitoring potentially toxic transformation products. The relative persistence of hopanoid and sterane biomarkers provided additional context for interpreting hydrocarbon compositional changes. Furthermore, the observed associations among microbial community composition, predicted functional potential, and metabolite profiles suggest that hydrocarbon transformation was supported by a diverse microbial community with complementary metabolic capabilities. This study integrated taxonomic profiles, predicted functional attributes, and metabolite data to provide a comprehensive framework for investigating hydrocarbon transformation during crude oil sludge co-composting. Beyond conventional bulk removal metrics, the metabolite-resolved approach offers additional insight into remediation performance and possible residual ecological risk, with potential applications in amendment selection, bioaugmentation strategy development, and environmental management. Overall, the findings contribute to a detailed assessment of microbial and biochemical dynamics across manure-amended composting systems and provide practical guidance for process optimization and large-scale bioremediation applications.

## Figures and Tables

**Table 1:** Experimental treatment setup and sample coding system used in the co-composting microcosm study.

Sample Code	Treatment Description	Manure Type
DCM	Solvent extraction for samples (dichloromethane)	None
ACE	Solvent blank GC/MS control (acetone)	None
CNT	Unamended control (soil + oil sludge, no manure)	None
SO	Soil-only control (no oil, no manure)	None
OIL	Crude oil sludge (oil-contaminated control)	None
CO1, CO2, CO3	Cow-manure-amended co-compost (replicates)	Cow
H1, H2, H3	Horse-manure-amended co-compost (replicates)	Horse
P11, P12, P13	Pig/swine-manure-amended co-compost (replicates)	Pig
PO1, PO2, PO3	Poultry-manure-amended co-compost (replicates)	Poultry
MIX COM	Mixed manure co-compost → all four manures together	Mixed

**Table 2:** Characteristics of the garden soil substrate.

Parameter	Value	Parameter	Value
Sand (% wt)	61.3	Cr (mg kg^−1^)	121.7
Silt (% wt)	21.3	Pb (mg kg^−1^)	31.91
Clay (% wt)	9.3	Ni (mg kg^−1^)	10.13
Texture	Sandy loam	Cu (mg kg^−1^)	38.08
pH (H_2_O)	5.56	Zn (mg kg^−1^)	9.65
Total organic C (mg kg^−1^)	13.01	Mn (mg kg^−1^)	92.38
Total organic N (mg kg^−1^)	3.94	Fe (mg kg^−1^)	67.04
Total P (mg kg^−1^)	4.4	Co (mg kg^−1^)	2.45
Dry matter (% DM)	90.48	Mg (mg kg^−1^)	22.37
Moisture (% MC)	9.52	Water holding capacity (%)	32.62

**Table 3:** Physicochemical characteristics of the animal manures used as co-composting amendments. Values are the means of three replicates ± standard error.

Animal Manure	Extractable Organic C (mg kg^−1^)	Extractable Organic N (mg kg^−1^)	Extractable P (mg kg^−1^)	C:N	C:P
Pig	904 ± 84	50.6 ± 5.9	252 ± 29	17.9	3.59
Poultry	277 ± 63	49.2 ± 14.2	254 ± 14	5.6	1.09
Cow	109 ± 8	54.9 ± 5.9	46 ± 8	2.0	2.37
Horse	81 ± 3	52.7 ± 2.7	50 ± 2	1.5	1.62

**Table 4:** Ash mass (g) at the initial and end stages of composting (300 days).

Soil–Compost Mixture	Initial	End
Poultry	4.03 ± 0.21	4.08 ± 0.16
Cow	4.01 ± 0.19	4.01 ± 0.20
Pig	3.77 ± 0.15	3.78 ± 0.14
Horse	3.34 ± 0.04	3.36 ± 0.04
Control	4.04 ± 0.33	4.07 ± 0.32

There was no significant change in mineral fraction, showing that composting does not deplete soil mineral components.

**Table 5:** Alpha-diversity indices of microbial communities across treatments (16S rRNA, Illumina MiSeq).

Treatment	Observed OTUs	Chao1	Shannon (H′)	Simpson (1-D)
Poultry	774	1035.8	3.56	0.930
Horse	752	1185.5	2.83	0.850
Swine/Pig	775	982.2	4.27	0.975
Cow	959	1350.2	3.66	0.888
Control	967	1405.8	3.90	0.955

**Table 6:** Initial and residual concentrations and removal efficiencies of 18 PAHs after 300 days of co-composting.

PAH (Rings)	Initial (mg kg^−1^)	Poultry %	Horse %	Cow %	Pig %	Control %
Naphthalene (2)	98 ± 6.2	99.9	99.9	99.9	99.9	97.0
Acenaphthylene (3)	6.0 ± 0.8	93.8	96.0	91.9	96.2	84.0
Acenaphthene (3)	9.2 ± 1.2	97.3	99.9	99.2	99.9	86.8
Fluorene (3)	27 ± 3.1	96.7	99.9	99.6	99.9	84.0
Anthracene (3)	42 ± 4.6	99.5	99.9	99.4	99.9	97.3
Phenanthrene (3)	15 ± 2.1	76.1	99.4	98.9	99.6	70.2
Fluoranthene (4)	2.4 ± 0.6	89.1	92.4	80.3	93.7	60.5
Pyrene (4)	14 ± 1.1	93.2	94.5	78.9	97.9	77.0
Chrysene (4)	55 ± 4.2	97.3	97.8	95.8	98.8	81.7
Benzo[a]anthracene (4)	4.1 ± 1.2	71.8	74.3	36.5	79.9	35.3
Benzo[b]fluoranthene (5)	24 ± 2.7	95.9	95.8	95.1	96.3	91.6
Benzo[k]fluoranthene (5)	2.6 ± 0.3	59.4	62.9	69.3	51.9	51.9
Benzo[a]pyrene (5)	10.0 ± 1.8	82.7	82.4	74.8	82.8	72.5
Perylene (5)	5.1 ± 2.0	69.9	64.2	57.4	61.6	50.1
Indeno [1,2,3-cd]pyrene (6)	10 ± 2.9	70.6	85.2	74.4	86.7	57.1
Dibenzo[a,h]anthracene (5)	12 ± 1.9	39.7	74.8	54.9	81.7	39.8
Benzo[ghi]perylene (6)	9.4 ± 1.1	40.3	72.6	45.8	77.7	40.3
Benzo[e]acephenanthrylene (5)	3.9 ± 0.1	75.9	74.6	71.8	79.0	50.0
Mean removal	—	80.5	87.0	79.1	88.0	68.2

Residual concentrations and removal efficiencies are the means of three replicates; values in the manuscript table include ± standard error.

**Table 7:** Predicted abundance of key catabolic KEGG Orthologs (KOs) for hydrocarbon and aromatic compound degradation (PICRUSt2).

KO	Enzyme (Gene)	Pathway Role	Poultry	Horse	Swine/Pig	Cow	Control
K03381	Catechol 1,2-dioxygenase	Ortho ring-cleavage	81,220	38,267	52,480	27,394	49,022
K00446	Catechol 2,3-dioxygenase	Meta ring-cleavage	2517	1079	14,151	6215	20,316
K07104	Catechol dioxygenase	Ring-cleavage	8987	2274	30,139	38,307	29,822
K00448	Protocatechuate 3,4-dioxygenase α	β-ketoadipate branch	59,895	55,130	38,831	10,974	44,465
K00449	Protocatechuate 3,4-dioxygenase β	β-ketoadipate branch	62,047	59,397	38,867	11,323	44,486
K01856	Muconate cycloisomerase	Lower pathway	83,752	39,199	43,070	18,683	39,466
K01055	3-Oxoadipate enol-lactonase	Lower pathway	107,815	65,015	69,629	25,781	68,727
K00496	Alkane 1-monooxygenase (alkB)	Aliphatic oxidation	14,056	13,591	9703	37,264	23,965
K03379	Benzoate 1,2-dioxygenase	Aromatic activation	3029	2474	10,541	34,540	17,100
K05549	Benzoate/toluate 1,2-dioxygenase α	Aromatic activation	55,471	25,736	47,911	23,679	15,009
K05550	Benzoate/toluate 1,2-dioxygenase β	Aromatic activation	69,695	41,677	44,600	24,420	14,288
K00480	Salicylate hydroxylase	Naphthalene upper pathway	32,196	27,569	26,485	4896	37,918
K00481	Salicylate hydroxylase	Naphthalene upper pathway	43,036	37,438	43,546	8546	54,913
K00055	Aryl-alcohol dehydrogenase	Aromatic alcohol oxidation	7720	4663	6184	5593	8494

Values are PICRUSt2-predicted KO abundances (predicted gene copy number). These represent functional potential inferred from 16S rRNA phylogeny, not directly sequenced genes.

**Table 8:** Chemical class distribution of identified metabolites across experimental treatments.

Chemical Class	Representative Compounds	Occurrence Range (No. of Samples)	Biochemical Significance
n-Alkanes (C14–C36)	Tetradecane, hexadecane, eicosane, hexacosane, hexatriacontane	5–15	Petrogenic signature; aerobic terminal and subterminal oxidation substrates
Parent PAHs	Naphthalene, phenanthrene, anthracene, pyrene, chrysene, fluoranthene	8–15	Priority pollutants; aromatic ring-hydroxylation substrates
Alkylated PAHs	Methylnaphthalenes, methylphenanthrenes, methylpyrenes, dimethylchrysenes	10–17	Petrogenic markers; slower biodegradation kinetics than parents
Sulfur-heterocyclic PAHs	Dibenzothiophene, 1,7-dimethyldibenzothiophene, benzo[b]naphtho [2,3-d]thiophene	10–15	Petrogenic S-PAHs; desulfurisation and ring-cleavage substrates
Oxygenated PAH intermediates	1,4-Naphthoquinone, 2-hydroxyfluorene, phenanthrene-methanol, and benzenediols	3–12	Ring-dihydroxylation and catechol cleavage products; biodegradation progression markers
Phthalate esters	Diisooctyl phthalate, dibutyl phthalate, bis(2-ethylhexyl) phthalate	5–10	Matrix background and phthalate degradation pathway substrates
Hopanoids and steranes	17α,21β-28,30-Bisnorhopane, androstane, stigmastane	8–14	Conservative petrogenic biomarkers; biodegradation severity indices
Fatty acids and alcohols	1-Hexadecanol, Z-11-hexadecenal, oleic acid, stearic acid	10–17	Membrane lipids; β-oxidation products of alkane degradation
Organic acids	Butanedioic acid, propanoic acid, and oxalic acid esters	5–12	TCA cycle intermediates; catechol cleavage terminal products
Phenolic compounds	Phenol, p-cresol, phloroglucinol, xanthoxylin	4–15	Aromatic ring-cleavage intermediates; lignin decomposition products
Biphenyl/terphenyl derivatives	Biphenyl, o-terphenyl, 4-ethyl-1,1′:4′,1″-terphenyl	8–15	Biphenyl degradation pathway substrates and petrogenic indicators

**Table 9:** Priority unknown metabolite features recurring across ≥15 of 17 samples.

FEATURE ID	SAMPLES DETECTED	PRIORITY FOR MS/MS CONFIRMATION
Unknown 1–9	17/17	High
Unknown 10–11	17/17	High
Unknown 12–13	16/17	High
Unknown 18–19, 25	15/17	High
Unknown 32–33	15/17	High
Unknown 69–77	15/17	Moderate–High

**Table 10:** Identification of intermediate metabolites formed during crude-oil refinery waste-oil-sludge PAH co-composting by GC-MS. For each metabolite, the table reports the molecular formula, retention time, characteristic (quantifier) *m*/*z*, occurrence across the 17 treatments, NIST spectral library match (similarity index), assigned role in the catabolic pathway, and Metabolomics Standards Initiative (MSI) identification confidence level.

Metabolite Name	Molecular Formula	Retention Time (min)	Quantifier *m*/*z*	Occurrence (17)	NIST Similarity Index	Pathway Role	MSI Confidence Level
Oxygenated Ring Intermediates							
2-Hydroxyfluorene	C_13_H_10_O	18.24	180	3	882	Oxygenated ring intermediate (PAH mono-hydroxylation)	MSI Level 2
Octahydrophenanthrenemethanol	C_15_H_22_O	22.41	218	4	761	Oxygenated ring intermediate (partial ring reduction)	MSI Level 2
(2-Methyl-3-biphenylyl)methanol	C_14_H_14_O	19.87	198	2	793	Oxygenated ring intermediate (biphenyl hydroxylation)	MSI Level 2
Dihydro-1,4-naphthoquinone	C_10_H_8_O_2_	15.63	160	2	820	Oxygenated ring intermediate (quinone formation)	MSI Level 2
Short-Chain Organic Acids							
Butanedioic acid derivative	C_4_H_6_O_4_	8.92	118	14	961	Short-chain organic acid (TCA cycle substrate)	MSI Level 1
Propanoic acid	C_3_H_6_O_2_	5.14	74	11	955	Short-chain organic acid (lower-pathway product)	MSI Level 1
Oxalic acid	C_2_H_2_O_4_	4.38	90	9	943	Short-chain organic acid (terminal mineralisation product)	MSI Level 1
Fatty Alcohol							
1-Hexadecanol	C_16_H_34_O	24.17	242	6	972	Fatty alcohol (aliphatic oxidation channel)	MSI Level 1
Alkylated Polycyclic Aromatic Hydrocarbons (PAHs)							
1,1,4,5,6-Pentamethyl-2,3-dihydro-1H-indene	C_14_H_20_	21.55	188	15	910	Alkylated aromatic (recalcitrant PAH fraction)	MSI Level 2
1,3-Dimethylpyrene	C_18_H_14_	28.33	230	14	874	Alkylated PAH (high-MW aromatic)	MSI Level 2
Methylpyrene	C_17_H_12_	27.44	216	11	891	Alkylated PAH (high-MW aromatic)	MSI Level 2
Tetramethylnaphthalene	C_14_H_16_	17.22	184	11	903	Alkylated PAH (low-MW aromatic)	MSI Level 2
Sulfur-Heterocyclic PAHs (Dibenzothiophene Series)							
Dimethyldibenzothiophene (1,7-isomer)	C_14_H_12_S	25.61	212	11	812	Sulfur-heterocyclic PAH (DBT series)	MSI Level 2
Dimethyldibenzothiophene (2,7-isomer)	C_14_H_12_S	25.84	212	10	798	Sulfur-heterocyclic PAH (DBT series)	MSI Level 2
Benzo[b]naphtho [2,3-d]thiophene, 9,10-dihydro-7-methyl-	C_17_H_14_S	30.12	250	15	780	Sulfur-heterocyclic PAH (partially hydrogenated)	MSI Level 2
Phthalate Esters							
Diisooctyl phthalate	C_24_H_38_O_4_	32.55	149	6	930	Phthalate ester (plasticiser-derived substrate)	MSI Level 2
Dibutyl phthalate	C_16_H_22_O_4_	20.88	149	3	945	Phthalate ester (plasticiser-derived substrate)	MSI Level 1
Conservative Petroleum Biomarkers							
17α,21β-Bisnorhopane	C_28_H_48_	38.44	370	2	841	Conservative petroleum biomarker (non-degradable reference)	MSI Level 2
Sterane (C_27_)	C_27_H_48_	36.22	372	1	660	Conservative petroleum biomarker (non-degradable reference)	MSI Level 3
Nitrogen-Heterocyclic Metabolites							
(3,4,5,6-Tetrahydro-2H-[2,3′]bipyridinyl-1-yl)acetic acid hydrazide	C_11_H_16_N_4_O	22.09	220	1	710	Nitrogen-heterocyclic metabolite (cow manure-specific)	MSI Level 3

MSI Level 1 = confirmed against authentic standard or high-quality reference spectrum; MSI Level 2 = putative identification based on spectral library match; MSI Level 3 = tentative identification (low similarity index); flagged for targeted MS/MS verification.

**Table 11:** Microbial consortium composition and endogenous biosurfactant capacity of active bacterial isolates. Isolate identity, co-compost origin, viable count, and biosurfactant status. All isolates were cultivated individually and combined at a 1:1 (*v*/*v*) ratio to form one homogenized, pre-adapted consortium. Biosurfactant rating (β): +++ = positive for oil displacement, drop-collapse, and emulsification.

#	Isolate (Strain Designation)	Closest Type Strain (16S rRNA Identity)	NCBI Accession	Co-Compost Sources	Viable Count (×10^4^ CFU mL^−1^)	Endogenous Biosurfactant Production
1	*Paenibacillus* sp. *strain CO15*	*Paenibacillus lautus NBRC 13380(T)*	MK854828.1	Cow (CO)	2.61	Producer (+++) oil-displacement, drop collapse & emulsification
2	*Cellulosimicrobium funkei strain PO181*	*Cellulosimicrobium funkei W6122*	MK854951.1	Poultry (PO)	3.10	Producer (+++) oil-displacement, drop collapse & emulsification
3	*Bacillus subtilis strain CO41*	*Bacillus subtilis JCM 1465*	MK854834.1	Cow (CO)	2.14	Producer (+++) oil-displacement, drop collapse & emulsification
4	*Micrococcus aloeverae strain MC10*	*Micrococcus aloeverae OAct925*	MK854858.1	Mix Compost (MC)	2.41	Producer (+++) oil-displacement, drop collapse & emulsification
5	*Rhodococcus equi strain CO20*	*Rhodococcus equi MDR-RE 2287*	MK854831.1	Cow (CO)	1.08	Producer (+++) oil-displacement, drop collapse & emulsification
6	*Streptomyces* sp. *strain PO62*	*Streptomyces* sp. *FZ42*	MK854943.1	Poultry (PO)	1.15	Producer (+++) oil-displacement, drop collapse & emulsification
7	*Pseudomonas stutzeri strain H131*	*Pseudomonas stutzeri WWvii23*	MK854849.1	Horse (H)	1.89	Producer (+++) oil-displacement, drop collapse & emulsification
8	*Lysinibacillus xylanilyticus strain PO49A*	*Lysinibacillus xylanilyticus XDB9*	MK854936.1	Poultry (PO)	2.06	Producer (+++) oil-displacement, drop collapse & emulsification
9	*Bacillus atrophaeus strain Pi1*	*Bacillus atrophaeus DSM 7264*	MK854883.1	Pig (Pi)	2.04	Producer (+++) oil-displacement, drop collapse & emulsification
10	*Burkholderia lata strain CT22*	*Burkholderia lata 383*	MK854978.1	Control (CT)	1.72	Producer (+++) oil-displacement, drop collapse & emulsification
11	*Paeniclostridium sordellii strain CO12*	*Paeniclostridium sordellii ATCC 9714*	MK854838.1	Cow (CO)	3.12	Producer (+++) oil-displacement, drop collapse & emulsification
12	*Pseudarthrobacter oxydans strain PO341*	*Pseudarthrobacter oxydans DSM 6612*	MK854924.1	Poultry (PO)	2.80	Producer (+++) oil-displacement, drop collapse & emulsification
13	*Sporosarcina* sp. *strain PO35*	*Sporosarcina* sp. *BYMS04*	MK854925.1	Poultry (PO)	1.40	Producer (+++) oil-displacement, drop collapse & emulsification
14	*Burkholderia paludis strain H93*	*Burkholderia paludis MSh1*	MK854855.1	Horse (H)	1.00	Producer (+++) oil-displacement, drop collapse & emulsification
15	*Sanguibacter* sp. *strain PO47*	*Sanguibacter* sp. *M2T8B10*	MK854933.1	Poultry (PO)	1.10	Producer (+++) oil-displacement, drop collapse & emulsification
16	*Sanguibacter soli strain CT121*	*Sanguibacter soli DCY22*	MK854971.1	Control (CT)	1.00	Producer (+++) oil-displacement, drop collapse & emulsification
17	*Bacillus thuringiensis strain OS21*	*Bacillus thuringiensis BST-122*	MK854877.1	Oil sludge (OS)	2.00	Producer (+++) oil-displacement, drop collapse & emulsification
18	*Gordonia* sp. *strain CT122*	*Gordonia* sp. *NB4-1Y*	MK854972.1	Control (CT)	2.00	Producer (+++) oil-displacement, drop collapse & emulsification
19	*Staphylococcus succinus subsp. succinus strain PO45*	*Staphylococcus succinus subsp. succinus DSM 14617*	MK854932.1	Poultry (PO)	1.90	Producer (+++) oil-displacement, drop collapse & emulsification
20	*Enterococcus mundtii strain CT10*	*Enterococcus mundtii ST4SA*	MK854970.1	Control (CT)	2.00	Producer (+++) oil-displacement, drop collapse & emulsification
21	*Burkholderia cenocepacia strain PO293a*	*Burkholderia cenocepacia ESS9*	MK854919.1	Poultry (PO)	1.79	Producer (+++) oil-displacement, drop collapse & emulsification
22	*Bacillus pumilus strain CO82II*	*Bacillus pumilus S1-10*	MK854835.1	Cow (CO)	2.00	Producer (+++) oil-displacement, drop collapse & emulsification
23	*Sphingomonas* sp. *strain H151*	*Sphingomonas* sp. *IMER-A1-23*	MK854857.1	Horse (H)	1.89	Producer (+++) oil-displacement, drop collapse & emulsification
24	*Bhargavaea* sp. *strain PO129*	*Bhargavaea* sp. *201802YP6*	MK854949.1	Poultry (PO)	2.00	Producer (+++) oil-displacement, drop collapse & emulsification
25	*Rhodococcus corynebacterioides strain PO141*	*Rhodococcus corynebacterioides DSM 20151*	MK854909.1	Poultry (PO)	1.50	Producer (+++) oil-displacement, drop collapse & emulsification
26	*Rhodococcus hoagii strain H121*	*Rhodococcus hoagii DSSKP-R-001*	MK854848.1	Horse (H)	2.89	Producer (+++) oil-displacement, drop collapse & emulsification
27	*Microbacterium hominis strain CT55*	*Microbacterium hominis DSM 12509*	MK854980.1	Control (CT)	1.00	Producer (+++) oil-displacement, drop collapse & emulsification
—	*Escherichia coli strain W3110* (negative control)	*Escherichia coli W3110*	NC_007779	Lab strain—not co-compost-derived	ND	*Not applicable (negative control)*
Consortium (active bacterial isolates): 27 isolates pooled at equal volumes (1 mL each; 1:1, *v*/*v*). Biosurfactant producers = 27 (71%); producers span every source tier (cow, horse, poultry, pig, mix compost, control, oil sludge), showing a distributed surface-active capacity.						

**Table 12:** Time-course biosurfactant production and surface activity parameters during crude oil sludge biodegradation by the assembled bacterial consortium with and without exogenous Tween 80 (0.1% *w*/*v*) supplementation. Incubation conditions: 30 °C, 150 rpm, crude oil sludge as sole carbon source. Cell-free supernatant collected by centrifugation at 8500× *g*, 4 °C, 10 min. Values represent mean ± SD (*n* = 3).

Day	OD_600_		Surface Tension (mN/m)		E_24_ (%)		Biosurfactant (mg/L)		Drop Collapse		Disp. Area (cm^2^)	
	Cons.	TW80	Cons.	TW80	Cons.	TW80	Cons.	TW80	Cons.	TW80	Cons.	TW80
0	0.05 ± 0.01	0.05 ± 0.01	72.00 ± 0.26	43.20 ± 0.32	0.00 ± 0.00	20.50 ± 0.79	0.0 ± 0.0	0.0 ± 0.0	−	++	0.00	1.77
1	0.09 ± 0.01	0.12 ± 0.01	70.50 ± 0.42	42.00 ± 0.42	3.20 ± 0.42	22.80 ± 1.06	8.0 ± 1.3	12.0 ± 1.9	−	++	0.07	2.01
2	0.28 ± 0.02	0.35 ± 0.03	62.80 ± 0.79	39.50 ± 0.63	12.50 ± 1.16	30.20 ± 1.48	35.0 ± 3.4	42.0 ± 4.2	+	++	0.50	2.54
3	0.65 ± 0.03	0.72 ± 0.04	50.20 ± 1.06	35.80 ± 0.79	28.80 ± 1.85	40.50 ± 1.85	108 ± 7.9	118 ± 8.5	+	+++	1.13	2.84
4	1.22 ± 0.04	1.30 ± 0.05	39.50 ± 0.95 *	32.20 ± 0.53	42.50 ± 2.12	50.80 ± 1.69	225 ± 11.6	240 ± 12.7	++	+++	2.01	3.14
5	1.58 ± 0.04	1.65 ± 0.04	33.80 ± 0.63 ^a^	30.50 ± 0.42 ^a^	55.20 ± 1.85 ^a^	58.50 ± 1.48 ^a^	335 ± 13.2	350 ± 14.8	++	+++	2.69	3.46
6	1.75 ± 0.03	1.80 ± 0.04	31.00 ± 0.48 ^a^	29.20 ± 0.37 ^a^	62.00 ± 1.48 ^a^	64.20 ± 1.32 ^a^	395 ± 10.6	415 ± 11.6	+++	+++	3.14	3.63
7	1.82 ± 0.03	1.88 ± 0.03	29.80 ± 0.37 ^a^	28.80 ± 0.26 ^a^	64.35 ± 1.32 ^a^	66.00 ± 1.16 ^a^	420 ± 9.5	440 ± 10.6	+++	+++	3.43	3.73

Cons. = consortium without Tween 80; +Tw80 = consortium + 0.1% (*w*/*v*) Tween 80; E_24_ = emulsification index after 24 h; Drop collapse scored as: − (beaded), + (slight spreading), ++ (partial collapse), +++ (complete collapse). * = surface tension crossed the 40 mN/m biosurfactant-indicative threshold. Superscript a = no significant difference between conditions (*p* > 0.05, two-way ANOVA with Tukey HSD post-hoc test).

## Data Availability

Data associated with this study have been deposited in the NCBI Sequence Read Archive (SRA) under BioProject ID PRJNA794053. All other original data are presented in this study. Further inquiries should be directed to the corresponding author.

## Abbreviations

The following abbreviations are used in this manuscript:

COWS	Crude oil refinery waste sludge
TPH	Total petroleum hydrocarbon
PAH(s)	Polycyclic aromatic hydrocarbons(s)
OPAH(s)	Oxygenated polycyclic aromatic hydrocarbon(s)
GC-MS	Gas chromatography–mass spectrometry
LC-MS	Liquid chromatography–mass spectrometry
MS/MS	Tandem mass spectrometry
KEGG	Kyoto Encyclopedia of Genes and Genomes
16S rRNA	16S ribosomal RNA gene
OTU(s)	Operational taxonomic unit(s)
ASV(s)	Amplicon sequence variant(s)
PICRUSt2	Phylogenetic Investigation of Communities by Reconstruction of Unobserved States, v2
KO	KEGG Orthology
NSTI	Nearest Sequenced Taxon Index
MSI	Metabolomics Standards Initiative
NIST	National Institute of Standards and Technology
PCA	Principal component analysis
EPA	United States Environmental Protection Agency
ICP-OES	Inductively coupled plasma optical emission spectrometry
TOC	Total organic carbon
WHC	Water-holding capacity
MSM	Mineral salts medium
MSA	Mineral salts agar
PBS	Phosphate-buffered saline
PCR	Polymerase chain reaction
qPCR	Quantitative polymerase chain reaction
DBT	Dibenzothiophene
TCA	Tricarboxylic acid (cycle)
E_24_	Emulsification index (24 h)
DCM	Dichloromethane
ACE	Acetone (solvent blank control)
DNA	Deoxyribonucleic acid
RHD	Ring-hydroxylating dioxygenase
DDH	cis-Dihydrodiol dehydrogenase
C12O/C23O	Catechol 1,2-dioxygenase/Catechol 2,3-dioxygenase
AlkB	Alkane 1-monooxygenase
CYP153	Cytochrome P450 (CYP153 family) alkane hydroxylase
Dsz/4S	Dibenzothiophene desulfurisation (4S) pathway enzymes
GNPS	Global Natural Products Social molecular networking
SILVA	SILVA ribosomal RNA reference database
BLAST	Basic Local Alignment Search Tool
NCBI	National Center for Biotechnology Information
UCHIME	Chimera-detection algorithm
ngsShoRT	Next-generation sequencing short-read trimmer
RDKit	Open-source cheminformatics toolkit
PVC	Polyvinyl chloride
